# Pre-Holocene Origin for the *Coronopus navasii* Disjunction: Conservation Implications from Its Long Isolation

**DOI:** 10.1371/journal.pone.0159484

**Published:** 2016-07-27

**Authors:** Sara Martín-Hernanz, Alejandro G. Fernández de Castro, Juan Carlos Moreno-Saiz, Virginia Valcárcel

**Affiliations:** 1 Department of Biology (Botany), Universidad Autónoma de Madrid, Madrid, Spain; 2 Department of Biodiversity and Conservation, Real Jardín Botánico, CSIC, Madrid, Spain; National Cheng-Kung University, TAIWAN

## Abstract

Integration of unexpected discoveries about charismatic species can disrupt their well-established recovery plans, particularly when this requires coordinate actions among the different governments responsible. The Critically Endangered *Coronopus navasii* (Brassicaceae) was considered a restricted endemism to a few Mediterranean temporary ponds in a high mountain range of Southeast Spain, until a new group of populations were discovered 500 km North in 2006. Ten years after this finding, its management has not been accommodated due to limited information of the new populations and administrative inertia. In this study, DNA sequences and species distribution models are used to analyse the origin of the *C*. *navasii* disjunction as a preliminary step to reassess its recovery plan. Molecular results placed the disjunction during Miocene-Pleistocene (6.30–0.49 Mya, plastid DNA; 1.45–0.03 Mya, ribosomal DNA), which discards a putative human-mediated origin. In fact, the haplotype network and the low gene flow estimated between disjunct areas suggest long-term isolation. Dispersal is the most likely explanation for the disjunction as interpreted from the highly fragmented distribution projected to the past. Particularly, a northward dispersal from Southeast is proposed since *C*. *navasii* haplotype network is connected to the sister-group through the southern haplotype. Although the reassessment of *C*. *navasii* conservation status is more optimistic under the new extent of occurrence, its long-term survival may be compromised due to the: (1) natural fragmentation and rarity of the species habitat, (2) genetic isolation between the two disjunct areas, and (3) northward shift of suitable areas under future climate change scenarios. Several *ex-situ* and *in-situ* conservation measures are proposed for integrating Central East Spanish populations into the on-going recovery plan, which still only contemplates Southeast populations and therefore does not preserve the genetic structure and diversity of the species.

Rare species are not invariably threatened with imminent extinction. However, those species that are threatened are almost invariably rare.

− Kevin Gaston

## Introduction

Selection of flagship species is key for effective biodiversity conservation management since they attract the attention of the public and policy administrators. Flagships species normally are endemic taxa restricted to a particular ecosystem, being mammals and birds the most common candidates to play this role [1], with the occasional exception of some plants [2]. The benefits of flagships often go beyond their own conservation improvements since they can also be umbrella species under which protection other species or habitats are also preserved [1].

Well-known threatened species are easy targets of well-supported conservation status assessments and more likely to become flagship species. The more conservation attention received, the more probable new discoveries occurred and resulted in improving the conservation status assessment. Reassessments in the conservation status force the adaptation of the conservation strategy to accommodate management actions. However, the implementation of new measures often faces researchers’ or politicians’ resistance delaying integration in the on-going recovery plans [3]. This is the case of *Coronopus navasii* Pau (Brassicaceae), one of the most charismatic species within the broad spectrum of the Spanish plant conservationism [4]. It is an endemic species that has been legally protected under the maximum risk category since the first National Catalogue of Endangered Species published in 1982 [5]. *Coronopus navasii* inhabits temporarily flooded clay ponds in xeric environments at high altitudes where it is locally abundant and dominant [5, 6]. Apart from its local abundance, *C*. *navasii* satisfies the two remaining features described by Rabinowitz [7] to be considered a rare species, it: (1) shows a high habitat specificity, and (2) was known from just a single metapopulation in the *Sierra de Gádor* mountain range (Andalusia, Southeast Spain). The species is not only a rare plant but it is under decline [8], legitimate condition to be considered as threatened [9]. Population size has been decreasing since the first censuses in the 70s and its local populations suffer from extinctions and severe demographic oscillations [5, 6]. The species decline has been associated to climate change and human impacts such as over-grazing, plowing or the practice of off-road driving. Indeed, *C*. *navasii* is catalogued under the ‘Critically Endangered’ IUCN category in both Regional and National Red Lists [8, 10] as well as ‘in Danger of Extinction’ in the corresponding legal catalogues of protected flora [5, 11]. It is also included in the Bern Convention [12] and as a priority species in the European Habitats Directive [13]. The species is also protected through its habitat since Mediterranean temporary ponds are considered priority habitats belonging to the European Natura 2000 network (Natura 2000 code 3170). Within this legal context, an integrated conservation program was developed on the species and its habitat including *in situ*, *ex situ* and legal measures. As a result, a range of *ex situ* conservation techniques has already been done [14] including the implementation of population reinforcements [5, 15].

**Fig 1:**
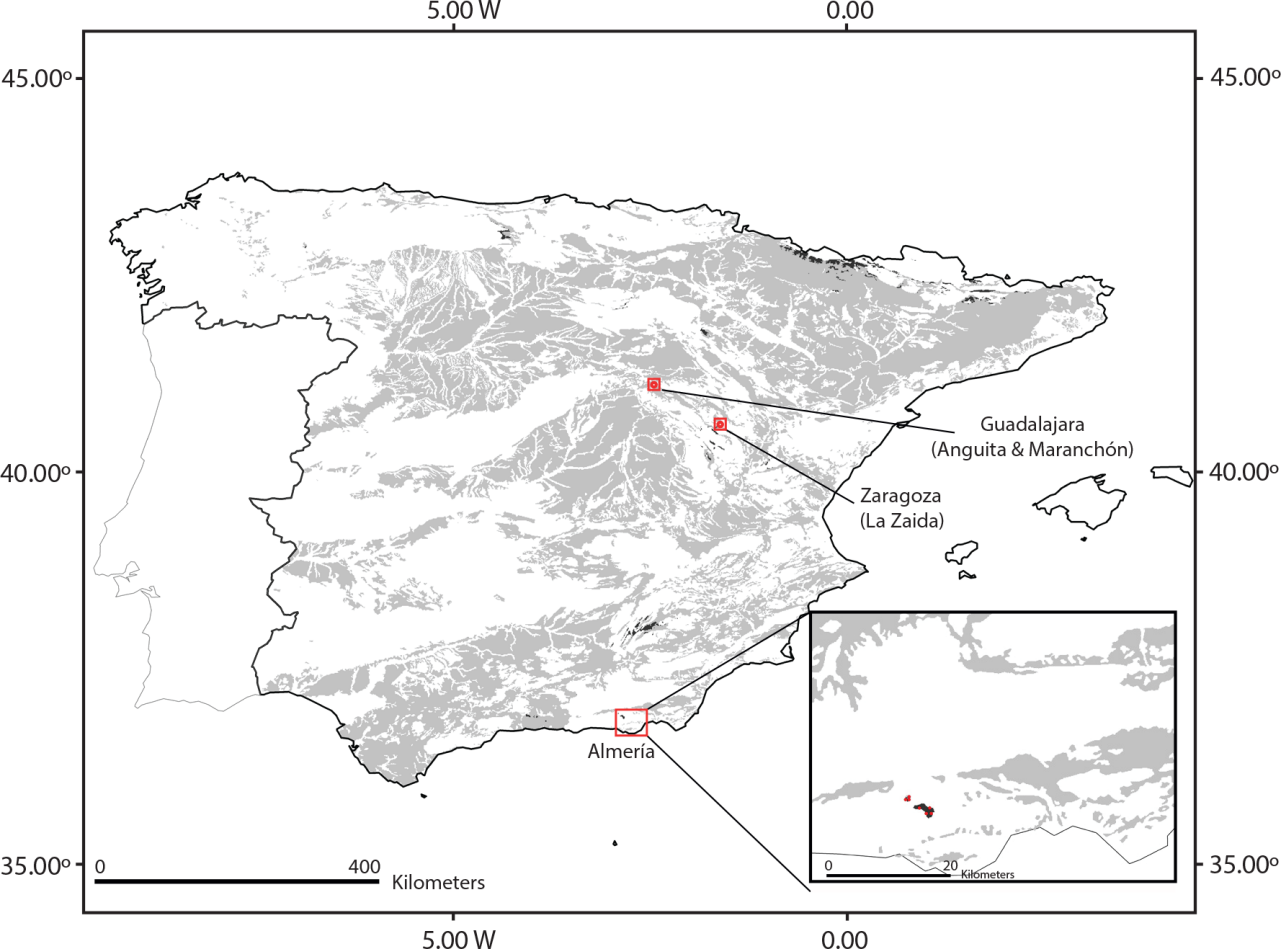
Distribution range of *Coronopus navasii*.

Unexpectedly, a new population was discovered in 2004 around 500 km North of *Sierra de Gádor*, in a similar habitat at the continental plateaus of the *Sistema Ibérico*. This population (hereafter called ‘*Anguita* population’, Guadalajara, Central East Spain; Fig 1) was discovered by López-Jiménez and García-Muñoz [16] in a small pool used as a watering hole. These authors suggested long distance dispersal (LDD) as the most plausible explanation for this disjunction between *Sierra de Gádor* and *Sistema Ibérico*, and proposed birds as the likely dispersal vector based on the epizoochory described for the species [5, 17]. The authors discarded vicariance as a plausible explanation due to the natural scattered occurrence of the ponds where *C*. *navasii* appears.

Interestingly, two new populations were found in the summer of 2014 in other localities at the *Sistema Ibérico* (Fig 1). One of these recently discovered populations (hereafter called ‘*Maranchón* population’) is located barely 16 km east of the *Anguita* population in a similar temporary pond (Julián García-Muñoz, *pers*. *com*.). The second one (hereafter called ‘*Zaida* population’) is in Zaragoza, c 100 km east of *Anguita* and *Maranchón* populations, where it occupies the temporary flooded shoulders of a farming path [18].

**Fig 2:**
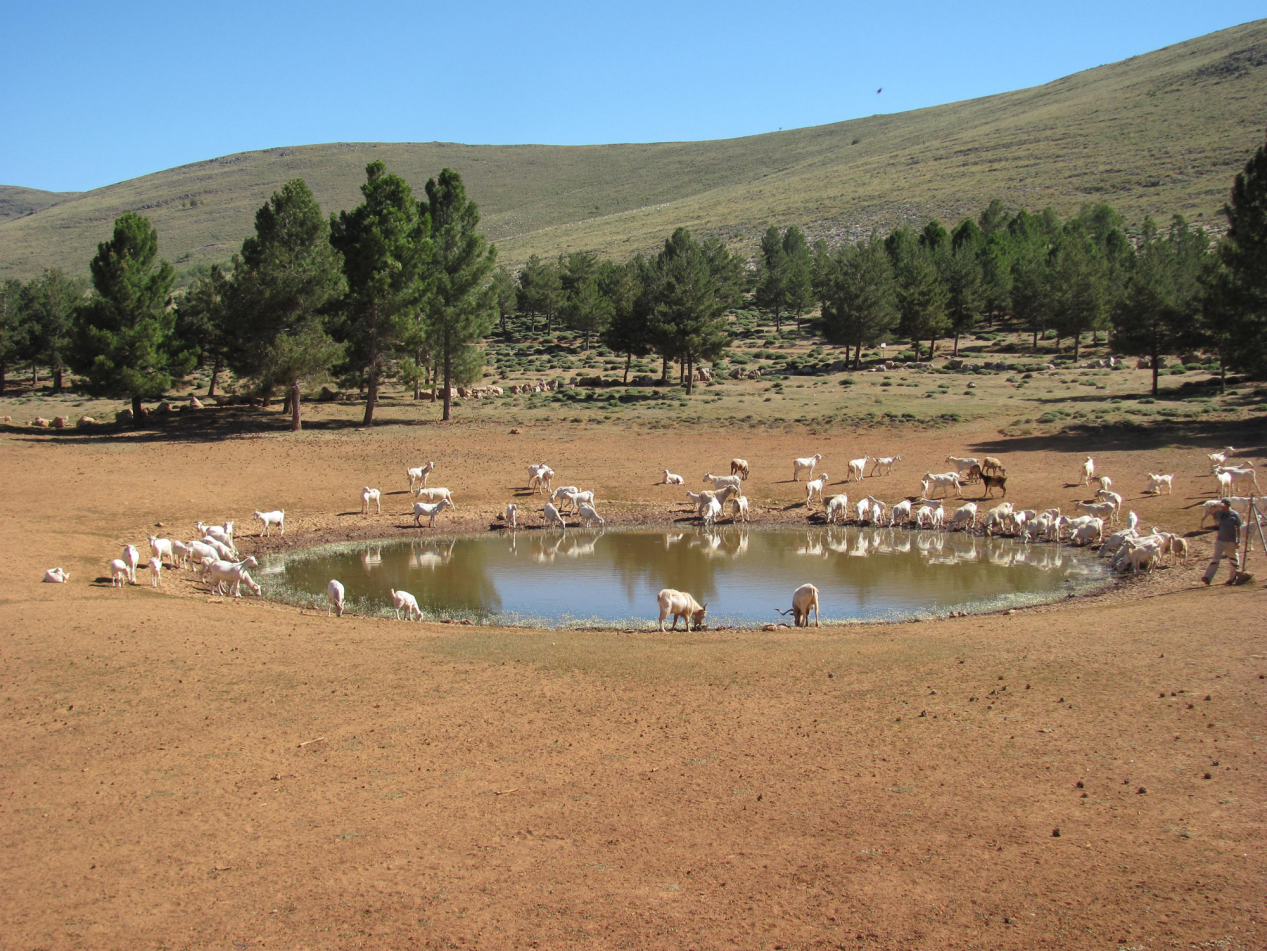
Natural habitat of *Coronopus navasii* (Temporary Mediterranean pond). *Sabinar* pond (*Sierra de Gádor*).

Mediterranean temporary ponds, like the ones where *C*. *navasii* occurs, are shallow waterholes flooded in winter and dry out by the beginning of summer. These ponds occur on superficial depressions over impermeable grounds. The degree of substrate impermeability and depression slope are key factors that control water level variability at a local scale [19]. However, rainfall is also a critical factor that determines flooding duration as well as size, depth and shape of ponds [19]. Every year, the hemicryptophyte *C*. *navasii* (S1 Fig) produces new aboveground shoots, leaves and flowers in the first line of vegetation as water drains. Its seeds fall by barochory after which an eventual secondary dispersal mechanism may happen by epizoochory. Fallen seeds are mixed into the mud and may get stuck to animals’ paws as they approach the pond to drink water [5]. The ponds where *C*. *navasii* grows have been traditionally used as natural watering holes for livestock supply (Fig 2) due to the impermeability of the clays [20, 21]. Because of this, shepherds may have probably favored these ponds to provide water for their flock during the long and dry summer. Given the recent discovery of the three distant northern populations in otherwise highly accessible localities related to traditional pastoral uses, coupled with their reduced population sizes, we could not help but wonder if the origin of these populations was human-mediated. This hypothesis is plausible since ‘transhumance’ has been a traditional land use in Spain until recent years [22]. This practice consisted in the regular displacement of cattle by shepherds to take advantage of spatial-temporal variations in land productivity associated to seasonal climates such as the Mediterranean one. Transhumance has a long history in the Iberian Peninsula, with some traces in pre-Roman times and its peak during the Middle Ages, when over 3 million *merino* sheep moved through the country going over up to 800 km distance [23]. The role of transhumance on plant dispersal has been experimentally evaluated revealing that seeds can be transported several hundreds of kilometers attached to hooves of transhumant sheep, even in plants with no adaptation to epizoochory such as *Plantago lagopus* L. [24]. For the particular case of *C*. *navasii*, transhumance-migration seems likely given that: (1) its individuals germinate in the first line of vegetation as water drains, (2) fruits phenology coincide with domesticated ungulates’ summer migrations and (3) ponds are used for flock drinking.

Despite *C*. *navasii* is one of the best documented cases of the Spanish endangered flora and one of the few with an integral conservation program already implemented, the recent finding of three distant populations changes the biogeographic scenario of the species questioning its conservation status and the efficiency of the on-going conservation program. Disclosing the evolutionary history that lead to *C*. *navasii* disjunction is essential to reassess the conservation status of the species and redefine its conservation strategy. In this study, we analyze DNA sequences and environmental data to evaluate whether the origin of the current disjunction of *C*. *navasii* was human-mediated (‘transhumance hypothesis’) or not. Ultimately, we seek to know if the occurrence of the species in Central East Spain is adventitious or the result of an old disjunction in order to reformulate the on-going species conservation plan to accommodate the new biogeographic scenario. To address these goals, we analyzed haplotype variation from six DNA regions in seven populations representing the two main disjunct areas of *C*. *navasii* (four populations from *Sierra de Gádor*, three populations from *Sistema Ibérico*). Additional datasets were obtained from previous studies (Malvidae, [25]; *Lepidium*, [26]) to provide a phylogenetic framework to estimate the divergence age for the origin of *C*. *navasii* and its disjunction. Finally, species distribution models were generated for past, present and future under different climate change scenarios. The specific goals were to: (1) test the monophyly of *C*. *navasii*, (2) explore the geographic structure of its haplotype variation, (3) disclose the temporal scenario for the origin of *C*. *navasii* and its disjunction, (4) describe an edaphic-climate niche modeling for the species at present time and evaluate niche performances under past and future climate scenarios, (5) propose conservation actions on the *Sistema Ibérico* populations and provide basic information to reevaluate the conservation status of the species.

## Materials and Methods

### Ethics statement

*Coronopus navasii* is an endangered species and no individual was collected. For this study, the minimum number of leaves was collected without compromising the survival of living plants. The environmental authorities of Andalusia, region where the species is protected under a recovery plan, granted the required permits for this study. Regional authorities from Aragón and Castilla-La Mancha are knowledgeable about this research and gave their formal approval to the collection of plant material in their respective areas of competence. This study did not require ethical approval.

### Study case

*Coronopus navasii* has between 8 and 10 local populations in *Sierra de Gádor* (Andalusia, Southeast Spain), occurring above 1,600 m.a.s.l. (Fig 1) and organized in a metapopulation dynamics [5]. Two local populations display a relatively large and constant population size: (1) *Cortijo de Caparidán* pond with around 37,000 individuals, and (2) *Sabinar* pond with nearly 1,200 individuals [5]. The remaining 6–8 local populations include fewer than 20 individuals each and are affected by inter-annual demographic fluctuations [5]. The *Sierra de Gádor* metapopulation is protected through: (1) its habitat since this mountain range is catalogued as a Site of Community Importance in the European Natura 2000 network (European code: ES6110008), (2) the conservation program of the species already in progress, and (3) a set of *in situ* conservation actions recently considered within the Recovery Plan for ‘Andalusian High Summit Species’ [27]. The three populations found in the surroundings of the *Sistema Ibérico* (Central East Spain) occur from 1,000 to 1,200 m.a.s.l. (Fig 1). Although, these altitudes cannot be considered high, the climatic conditions are extreme and subject to high seasonal fluctuation since this geographic area is a continental plateau considered a cold inland island in the Iberian Peninsula. The *Anguita* and *Maranchón* populations (Guadalajara; Fig 1) inhabit similar pond habitats as the ones described in the *Sierra de Gádor* metapopulation [16], whereas the *Zaida* population (Zaragoza; Fig 1) inhabits temporarily flooded sides of a country road that crosses a large and semi-permanent pond [18]. Three censuses have been performed in the *Anguita* population revealing a rather variable size ranging between < 100 reproductive individuals [16] and 800 [28]. Because of the recent discovery of the other two populations, only the respective initial censuses are available consisting in 450 reproductive individuals at *Maranchón* and 1,536 at *Zaida* [18]. In terms of management, the *Anguita* and *Maranchón* populations are protected through their habitat since they are located within a Site of Community Importance in the European Natura 2000 network (European code ES4240017). The *Zaida* population lacks any legal protection, although this locality is part of a Special Conservation Area under the EU Birds Directive (European code ES0000017).

### Molecular study

#### Field and lab work strategy

Fifty-four individuals from seven populations of *C*. *navasii* were sampled from the two disjunct areas where the species occurs (Fig 1, Table 1): 24 individuals from four local populations from *Sierra de Gádor* (Southeast Spain) and 30 from three populations of the continental plateaus of *Sistema Ibérico* (Central East Spain). The number of individuals per local population was ten except for two *Sierra de Gádor* locations (*Mercurio* and *Balsilla Alta*) where the population size was limited to two (Table 1). Leaf material was immediately stored in silica gel.

**Table 1:** List of studied material of Coronopus navasii used for the phylogenetic-based analyses, phylogeography and the niche modelling studies.

Individual number	Altitude	UTM	GenBank accession numbers						Plastid Hp
			*trn*H-*psb*A	*trn*S-*trn*G	*trn*L-*trn*F	*trn*T-*trn*L	*rps*16-*trn*Q	ITS	
***SIERRA DE GÁDOR (ALMERÍA*, *SOUTHEST SPAIN)***				*** ***	*** ***	*** ***	*** ***	*** ***	*** ***
**Sabinar** (E. Salmerón-Sánchez, F. Martínez-Hernández, F.J. Pérez-García and J.F. Mota-Poveda, 11-VII-2011)									
Individual 1	1835	30SWF1282	KM201532	KM201488	KM201483	KM201494	KM201513	KM201465	GAD
Individual 2	1835	30SWF1282	KM201533	KM201489	KU213763	KM201495	KM201514	KM201466	GAD
Individual 3	1835	30SWF1282	KU213670	KU213718	KU213764	KM201496	KM201515	KM201467	GAD
Individual 4	1835	30SWF1282	KU213671	KU213719	KU213765	KM201497	KM201516	KM201468	GAD
Individual 5	1835	30SWF1282	KU213672	KU213720	KU213766	KM201498	KM201517	KM201469	GAD
Individual 6	1835	30SWF1282	KU213673	KU213721	KU213767	KU213812	KU213843	KU213878	GAD
Individual 7	1835	30SWF1282	KU213674	KU213722	KU213768	KU213813	KU213844	KU213879	GAD
Individual 8	1835	30SWF1282	KU213675	KU213723	KU213769	KU213814	KU213845	KU213880	SAB8
Individual 9	1835	30SWF1282	KU213676	KU213724	KU213770	KU213815	KU213846	KU213881	GAD
Individual 10	1835	30SWF1282	KU213677	KU213725	KU213771	KU213816	KU213847	KU213882	GAD
**Cortijo de Caparidán** (E. Salmerón-Sánchez, F. Martínez-Hernández, F.J. Pérez-García and J.F. Mota-Poveda, 11-VII-2011)									
Individual 1	1600	30SWF0887	KM201534	KM201490	KM201484	KM201499	KM201518	KM201470	GAD
Individual 2	1600	30SWF0887	KM201535	KM201491	KU213772	KM201500	KM201519	KM201471	CAP2
Individual 3	1600	30SWF0887	KU213678	KU213726	KU213773	KM201501	KM201520	KM201472	GAD
Individual 4	1600	30SWF0887	KU213679	KU213727	KU213774	KM201502	KM201521	KM201473	GAD
Individual 5	1600	30SWF0887	KU213680	KU213728	KU213775	KM201503	KM201522	KM201474	GAD
Individual 6	1600	30SWF0887	KU213681	KU213729	KU213776	KU213817	KU213848	KU213883	GAD
Individual 7	1600	30SWF0887	KU213682	KU213730	KU213777	KU213818	KU213849	KU213884	GAD
Individual 8	1600	30SWF0887	KU213683	KU213731	KU213778	KU213819	KU213850	KU213885	GAD
Individual 9	1600	30SWF0887	KU213684	KU213732	KU213779	KU213820	KU213851	KU213886	GAD
Individual 10	1600	30SWF0887	KU213685	KU213733	KU213780	_	KU213852	KU213887	_
**Barranco del Mercurio** (J.C. Moreno-Saiz, F. Martínez-Hernández and S. Martín-Hernanz, 12-VI-2012)									
Individual 1	1720	30SWF1481	KU213688	_	KU213782	KM201504	KM201523	KM201475	MER1
Individual 2	1720	30SWF1481	KU213689	_	KM201485	KM201505	KM201524	KM201476	GAD
**Balsilla Alta** (F.J. Pérez-García and J.F Mota-Poveda, 21-VI-2012)									
Individual 1	2150	30SWF1484	KU213686	_	KU213781	KM201506	KM201525	KU213888	GAD
Individual 2	2150	30SWF1484	KU213687	KU213734	KM201486	KM201507	KM201526	KM201477	BAl2
***SISTEMA IBÉRICO (GUADALAJARA*, *ZARAGOZA*, *CENTRAL EAST SPAIN)***									
***Anguita*** (J.C. Moreno-Saiz and R. López-Huerta, 26-VI-2011)									
Individual 1	1250	30TWL54	KM201536	KM201492	KM201487	KM201508	KM201527	KM201478	IBE
Individual 2	1250	30TWL54	KM201537	KM201493	KU213783	KM201509	KM201528	KM201479	IBE
Individual 3	1250	30TWL54	KU213690	KU213735	KU213784	KM201510	KM201529	KM201480	IBE
Individual 4	1250	30TWL54	KU213691	KU213736	KU213785	KM201511	KM201530	KM201481	IBE
Individual 5	1250	30TWL54	KU213692	KU213737	KU213786	KM201512	KM201521	KM201482	IBE
Individual 6	1250	30TWL54	KU213693	KU213738	KU213787	_	KU213853	KU213889	_
Individual 7	1250	30TWL54	KU213694	KU213739	KU213788	_	KU213854	KU213890	_
Individual 8	1250	30TWL54	KU213695	KU213740	KU213789	KU213821	KU213855	KU213891	IBE
Individual 9	1250	30TWL54	KU213696	KU213741	KU213790	KU213822	KU213856	KU213892	ANG9
Individual 10	1250	30TWL54	KU213697	KU213742	KU213791	KU213823	KU213857	KU213893	IBE
**Maranchón** (S. Martín-Hernanz, V. Valcárcel, J. García and J.C. Moreno-Saiz, 25-VII-2014)									
Individual 1	1200	30TWL64	KU213698	KU213743	KU213792	KU213824	KU213858	KU213894	IBE
Individual 2	1200	30TWL64	KU213699	KU213744	KU213793	KU213825	KU213859	KU213895	IBE
Individual 3	1200	30TWL64	KU213700	KU213745	KU213794	KU213826	KU213860	KU213896	IBE
Individual 4	1200	30TWL64	KU213701	KU213746	KU213795	KU213827	KU213861	KU213897	IBE
Individual 5	1200	30TWL64	KU213702	KU213747	KU213796	KU213828	KU213862	KU213898	IBE
Individual 6	1200	30TWL64	KU213703	KU213748	KU213797	KU213829	KU213863	KU213899	IBE
Individual 7	1200	30TWL64	KU213704	KU213749	KU213798	_	KU213864	KU213900	_
Individual 8	1200	30TWL64	KU213705	KU213750	KU213799	KU213830	KU213865	KU213901	IBE
Individual 9	1200	30TWL64	KU213706	KU213751	KU213800	KU213831	KU213866	KU213902	IBE
Individual 10	1200	30TWL64	KU213707	KU213752	KU213801	KU213832	KU213867	KU213903	IBE
**Zaida** (V. Valcárcel, Á. Baltanás, and J.C. Moreno-Saiz, 22-IV-2015)									
Individual 1	1050	30TXL14	KU213708	KU213753	KU213802	KU213833	KU213868	KU213904	ZAI1
Individual 2	1050	30TXL14	KU213709	KU213754	KU213803	KU213834	KU213869	KU213905	ZAI1
Individual 3	1050	30TXL14	KU213710	KU213755	KU213804	KU213835	KU213870	KU213906	IBE
Individual 4	1050	30TXL14	KU213711	KU213756	KU213805	KU213836	KU213871	KU213907	ZAI4
Individual 5	1050	30TXL14	KU213712	KU213757	KU213806	KU213837	KU213872	KU213908	IBE
Individual 6	1050	30TXL14	KU213713	KU213758	KU213807	KU213838	KU213873	KU213909	ZAI1
Individual 7	1050	30TXL14	KU213714	KU213759	KU213808	KU213839	KU213874	KU213910	ZAI4
Individual 8	1050	30TXL14	KU213715	KU213760	KU213809	KU213840	KU213875	KU213911	IBE
Individual 9	1050	30TXL14	KU213716	KU213761	KU213810	KU213841	KU213876	KU213912	IBE
Individual 10	1050	30TXL14	KU213717	KU213762	KU213811	KU213842	KU213877	KU213913	ZAI4

Locality is provided indicating altitude, MGRS coordinates and voucher information. GenBank accession numbers are given for each of the DNA regions analyzed in each of the individuals. DNA haplotypes recovered with *trn*H-*psb*A, *rps*16-*trn*Q and *trn*T-*trn*L plastid regions are specified.

Total genomic DNA was isolated from leaf tissue using the extraction protocol DNAeasy Plant Mini Kiy (Qiagen, California, USA). Five plastid DNA regions as well as the nuclear ITS spacer were sequenced for all the 54 individuals of *C*. *navasii*, except for *trn*S*-trn*G and *trn*T*-trn*L from which only 51 and 50 individuals could be sequenced, respectively (Table 1). The forward and reverse primers used for the amplification of each of the six DNA regions as well as their respective PCR conditions were taken from the following studies: *psb*A and *trn*H [29] for *trn*H*-psb*A, *trn*S and *trn*G [30] for *trn*S*-trn*G, *trn*e and *trn*f [31] for *trn*L*-trn*F, *trn*a and *trn*b [31] for *trn*T*-trn*L, *trn*Q and *rps*16x1 [32] for *rps*16*-trn*Q, and 17SE and 26SE for the *nr*DNA ITS [33]. Amplifications were done in a MyClycer thermal cycler. PCR products successfully amplified were sequenced using the STABvida sequencing service (Big Dye Terminator v. 2.0, Applied Biosystem) and Secugen SL (Madrid, Spain). Sequences were edited using *Geneious* R8 [34]. All the DNA matrices were aligned using the windows interface MUSCLE [35] followed by further visual adjustments.

A comprehensive taxonomic sampling above the species-level was compiled to provide a robust phylogenetic framework to recover accurate estimates on divergence ages. Two datasets (nuclear ITS, plastid *trn*T-*trn*L) including 97 samples of *Lepidium s*.*l*. (*Cardaria*, *Coronopus*, *Lepidium*, *Stroganowia*, *Stubendorffia*, *Winklera*) plus *Hornungia petraea* as outgroup were provided by Klaus Mummenhoff based on Mummenhoff *et al*. 2009 (S1 Table) [26]. Four of the individuals of *C*. *navasii* from Southeast and Central East disjunct areas herein sequenced were added to both datasets. Each of the resulting two DNA matrices included 102 sequences (hereafter called ‘*Lepidium* nuclear matrix’ and ‘*Lepidium* plastid matrix’).

No feasible fossil record attributable to *Lepidium s*.*l*. is available. Consequently, a secondary calibration approach was needed in order to obtain calibration points to estimate the divergence age of *C*. *navasii* and its disjunction. To this purpose, 60 Brassicales and twelve Malvales sequences of the plastid *matK* gene were obtained from a previous study [25], as well as one from Crossosomatales and one from Huertales. All these 74 sequences were downloaded from the GenBank database (http://www.ncbi.nlm.nih.gov/genbank/). Since only one *Lepidium* sample was included in Hernández-Hernández *et al*. (2013) [25], five additional *Lepidium* species and one sample of *Coronopus squamatus* (Forssk.) Asch. were also taken from previous studies [36, 37, 38, 39], downloaded from GenBank and added to this dataset. As a result, a matrix including 79 *mat*K Malvidae sequences was built, using *Gerrardina* and *Tapiscia* as outgroup (hereafter called ‘Malvidae matrix’; S2 Table).

A haplotype-based phylogeographic study was conducted on the study species and its sister-group. Four plastid DNA regions (*trn*T-*trn*L, *trn*L-*trn*F, *rps*16-*trn*Q and *trn*H-*psb*A) were finally included in these analyses since the other two did not reveal any mutation (Table 1). To cover the sister-group in the phylogeographic approach, sequences of two plastid DNA regions (*trn*T-*trn*L, *trn*L-*trn*F) representing the two species of the sister-group of *C*. *navasii* (*C*. *squamatus* and *C*. *violaceus* (Munby) Kuntze, [26]) were taken from a previous study and downloaded from GenBank (S1 Table). As a result, two plastid DNA matrices were compiled. Firstly, the hereafter called ‘Mediterranean *Coronopus* clade matrix’ that included two plastid DNA regions (*trn*L-*trn*F and *trn*T-*trn*L) and 52 individuals: 50 of *C*. *navasii*, one of *C*. *squamatus*, and one of *C*. *violaceus* (Table 1 and S1 Table). Secondly, the hereafter called ‘*C*. *navasii* matrix’, which included three plastid DNA regions (*rps*16-*trn*Q, *trn*T-*trn*L and *trn*H-*psb*A) and 50 individuals of *C*. *navasii* (Table 1).

#### Divergence age estimates and phylogeographic analyses

Divergence age estimates were done as implemented in BEAST v.1.8.2 [40]. The simplest models of sequence evolution used (ITS1: K80+G, ITS2: K80+G, *trn*T-*trn*L: GTR+G) were selected as the best fitting models based on the Corrected Akaike Information Criterion (AICc) implemented in jModelTest 1.1b [41].

The molecular dating was performed in a two-step procedure due to the secondary calibration approach implemented (see above). The first step was conducted from the Malvidae matrix. Four fossil calibration points were selected for this first step based on a previous study ([25]; S2 Fig): (N1) a maximum bound age of 90.4 million years (Myr) and a minimum age of 88.5 Myr were applied to the divergence between *Luehea* clade and *Bixa* clade based on a fossil of *Dressiantha bicarpellata* [42], (N2) a maximum bound age of 61.9 Myr and a minimum age of 61.5 Myr were applied to the divergence between *Bretschneidera* and *Tropaeolum* based on a fossil of *Akania* sp. [43], (N3) a maximum bound age of 30.8 Myr and a minimum age of 29.2 Myr were applied to the divergence between *Thlaspi* and *Alliaria* based on a fossil of *Thlaspi primaevum* [43, 44], and (N4) 17 Myr and 16.3 Myr were applied as maximum and minimum bound ages to the divergence between *Capparis* and *Apophyllum*, based on a fossil of *Capparidoxylon holleisii* [43, 45]. Cross-validation [46] was conducted to check for congruence between the four fossils selected. Variation in the sum of the squared differences (SS) of the age recovered in the molecular estimate for a given node and its respective fossil age was computed when comparing the molecular estimates obtained using each of the four fossils as a single calibration point (S3 Fig). *Capparidoxylon* is the fossil with the greatest SS value, i.e. the one that provides the biggest deviation between molecular estimates and fossil ages, followed by *Thlapsi* (S3 Fig). However, removal of the most deviant fossil (*Capparidoxylon*) did not result in a significant difference in the variance (F = 2.09, d.f. = 11, *P* = 0.4). Therefore, the four fossils were used for this first step of the molecular dating. In the second step, three well-supported nodes recovered in the Malvidae chronogram (S2 Fig) were selected as secondary calibration points to reconstruct divergence age estimates within *Lepidium s*.*l*. Normal prior distributions were used to calibrate the divergence between the following nodes (S4 Fig): (N1) *Lepidium pedicellosum* clade and the remaining *Lepidum s*.*l*. (19.5 ± 3.0 Myr), (N2) *L*. *perfoliatum* and the *L*. *rigidum—L*. *campestre* clade with (12.35 ± 2.5 Myr), and (N3) *L*. *campestre* subclade and *L*. *hirtum* subclade (mean = 8.7 ± 1.5 Myr). For the plastid analysis, the Mediterranean *Coronopus* clade was constrained. Uncorrelated lognormal model distribution and the best fitting models selected by jModeltest were used. One hundred million generations were run, sampling every 10,000^th^ tree. Burn-in was determined based on the Likelihood convergence screened with Tracer 1.4. [47]. Trees retrieved before reaching convergence were accordingly discarded. Tree topologies obtained from the BEAST analyses of *Lepidium* nuclear and plastid matrices were compared by the Approximately Unbiased (AU) test [48], as implemented in Treefinder [49]. 10^5^ replicates were performed and hypothesis rejection was set at a 0.05 threshold. Bayes Factors (BF; [50, 51] were also estimated by using the stepping-stone sampling implemented in BEAST to provide additional test topology. The BF analysis allows comparing the strength of evidence between a reference model (H0) and an alternative model (Hi) given the data. The stepping-stone method provides more accurate marginal likelihood estimates than the harmonic mean method [52, 53]. Selection of the best competing hypotheses against the H0 is based on the log BF (LBF = 2loge (BF)) and according to Kass and Raftery’s interpretation (Positive evidence, 2loge(BF) = 2–6; Strong evidence, 2loge(BF) = 6–10; Very Strong evidence, 2loge (BF) > 10 [50]). Positive values of the LBF indicate preference for H1, while negative values favor the H0.

Plastid haplotype networks were obtained using Statistical Parsimony [54] as implemented in TCS 1.21 [55] both for Mediterranean *Coronopus* clade and *C*. *navasii* matrices. The maximum number of differences among haplotypes, as a result of single substitutions, was calculated with 95% confidence limits. Gaps were treated as missing data for the analysis of the *Coronopus* clade matrix to preserve the connection of the outgroup network to the one of *C*. *navasii*, whereas missing data were treated as the fifth character for the *C*. *navasii* matrix to include indel information in the detection of haplotypes.

#### Gene flow and genetic differentiation estimates

Two analyses were conducted to account for the current gene flow and migration rates between the Southeast and Central East groups of populations. Firstly, an analysis of molecular variance (AMOVA) was performed with software Arlequin 3.0 [56]. Secondly, software dnaSP was used to obtain both genetic differentiation estimators (FST and GST) and the number of migrants (Nm) estimated with Hudson et al. method [57].

### Species distribution modeling

#### Calibration and validation datasets

A dataset composed by the sampled populations spanned to 14 presences at 1 km^2^ grid resolution. A dataset of 1,000 pseudo absence points was randomly generated, preventing them to fall within 1 km of *C*. *navasii* occurrence cells. These set of presence and pseudo absence points were used to calibrate the models.

#### Predictor variables

Bioclimatic variables for current conditions were obtained from Worldclim project [58] at 30 seconds resolutions. The eight *a priori*, most meaningful species distribution models (SDMs) were selected from the 19 bioclimatic variables available (Table 2). Two topographic predictors were incorporated: Slope was derived in ArcGIS 9.3 [59] from a high resolution DEM (20 meters) available from the National Geographic Center and rescaled to match climate layers resolution. Topographic index was developed with ArcInfo routines available at Nicklaus Zimmerman website (http://bit.ly/1nSEhoO). The novel climate scenarios available in the Worldclim project [58] downscaled from the recent 5^th^ report of IPCC [60] were used to project calibrated models for years 2050 and 2070. HadGEM2-ES was the Global Circulation Model selected, as it accounted for the whole range of Representative Concentration Pathways (RCP) of greenhouse gases concentrations considered by the IPCC. Among them, we selected 2.6, 6.0 and 8.5 RCPs. For past climatic conditions, bioclimatic variables for the Last Interglacial period (LIG, 120–140 kilo years (ky)) and CCSM model for Last Glacial Maximum (LGM, 21 ky) were used from Worldclim.

**Table 2:** Contribution to variance of each selected variable for each model and hierarchical partitioning approach.

**Variable description**	**ENSEMBLE**	**GLM**	**GBM**	**CTA**	**ANN**	**MARS**	**RF**	**HP**
Annual Mean Temperature	0.607	0.913	0.307	0.211	0.274	0.918	0.139	22.45
Temperature Seasonality	0.097	0	0.297	0	0.185	0.00	0.287	14.84
Mean Temperature of Wettest Quarter	0.007	0	0.085	0.783	0.002	0.01	0.186	12.48
Mean Temperature of Driest Quarter	0.026	0.56	0.008	0	0	0.00	0.002	0.28
Annual Precipitation	0.044	0	0.129	0	0.081	0.120	0.056	6.40
Precipitation of Wettest Month	0.049	0	0	0	0	0.361	0.128	22.40
Precipitation of Wettest Quarter	0.018	0	0.006	0	0	0.42	0.027	4.57
Precipitation of Driest Quarter	0.048	0	0.001	0	0	0.116	0.001	6.19
Slope: Maximum rate of change in height value from each map cell to its neighbors	0.050	0	0.005	0	0.274	0.121	0	6.21
TPI: Topographic exposure at various spatial scales, hierarchically integrated into a single grid	0.00	0.483	0.041	0	0	0	0	4.21

Variables description and abbreviation are provided. Models are abbreviated as follows: Generalized linear model (GLM), boosted regression trees (GBM), classification tree analysis (CTA), artificial neural network (ANN), multiple adaptive regression splines (MARS), random forest (RF) and hierarchical partitioning (HP).

#### Model workflow

Niche model analysis was conducted with Biomod2 package [61] implemented in R software [62]. The chosen models were stepwise generalized linear models (GLM), boosted regression trees (GBM), classification tree analysis (CTA), artificial neural network (ANN), multiple adaptive regression splines (MARS) and random forests (RF). The ensemble modeling technique implemented in Biomod2 was utilized to build a single prediction. Because of the limited presence data available, the procedure was repeated 100 times splitting data into 20% and 80% for evaluation and calibration, respectively. One hundred absences were randomly selected from the total pool for each run and weighted to match the number of presences. One hundred permutations were run for each model to assess variable importance as implemented in Biomod2. A final ensemble model was built with all the runs with AUC > 0.7. Output suitability maps were crossed with a soil layer of Iberian Peninsula in order to account only for areas where clay soils were present. Complementarily, hierarchical partitioning approach [63] was conducted with the hier.part package [64] in R software to account for a linear method based on Ordinary Least Squares. In order to assess the changes in range sizes (i.e., changes in total potential distribution area), the presence-absence distribution maps were calculated from suitability maps from the threshold that maximized the AUC score. Changes in range sizes were calculated as the percentage of lost or gained cells with respect to present period.

For past climate conditions, pure climate suitability models were calibrated under present climate conditions and projected into the past climatic scenarios, as no topographic information for the past is available. For the same reason, output maps were not crossed with clay soil layers.

## Results

### Molecular study

#### Divergence age estimates

The Malvidae matrix is 1,667 pair of bases (bp) length with a total of 1,106 variable characters of which 751 are potentially parsimony-informative. The *Lepidium* nuclear matrix is 493 bp length with a total of 300 variable characters of which 184 are potentially parsimony-informative. The *Lepidium* plastid matrix is 668 bp length with a total of 203 variable characters of which 66 are potentially parsimony-informative. The topology of the BEAST chronogram resulting from the Malvidae matrix (S2 Fig) is mostly congruent with the one obtained by Hernández-Hernández et al. (2013) [25] except for between-genera relationships within the *Bixa* clade (S2 Fig). The age inferred for the three nodes selected as calibration points for the second step are as follow (S2 Fig): (N1) 27.74 Mya—12.72 Mya for the early divergence of *Lepidium*, (N2) 19.42 Mya—6.36 Mya for the divergence between *L*. *perfoliatum* and *Cardaria draba* clade, and (N3) 15.12 Mya—3.79 Mya between *Cardaria draba* and *L*. *campestre*.

**Fig 3:**
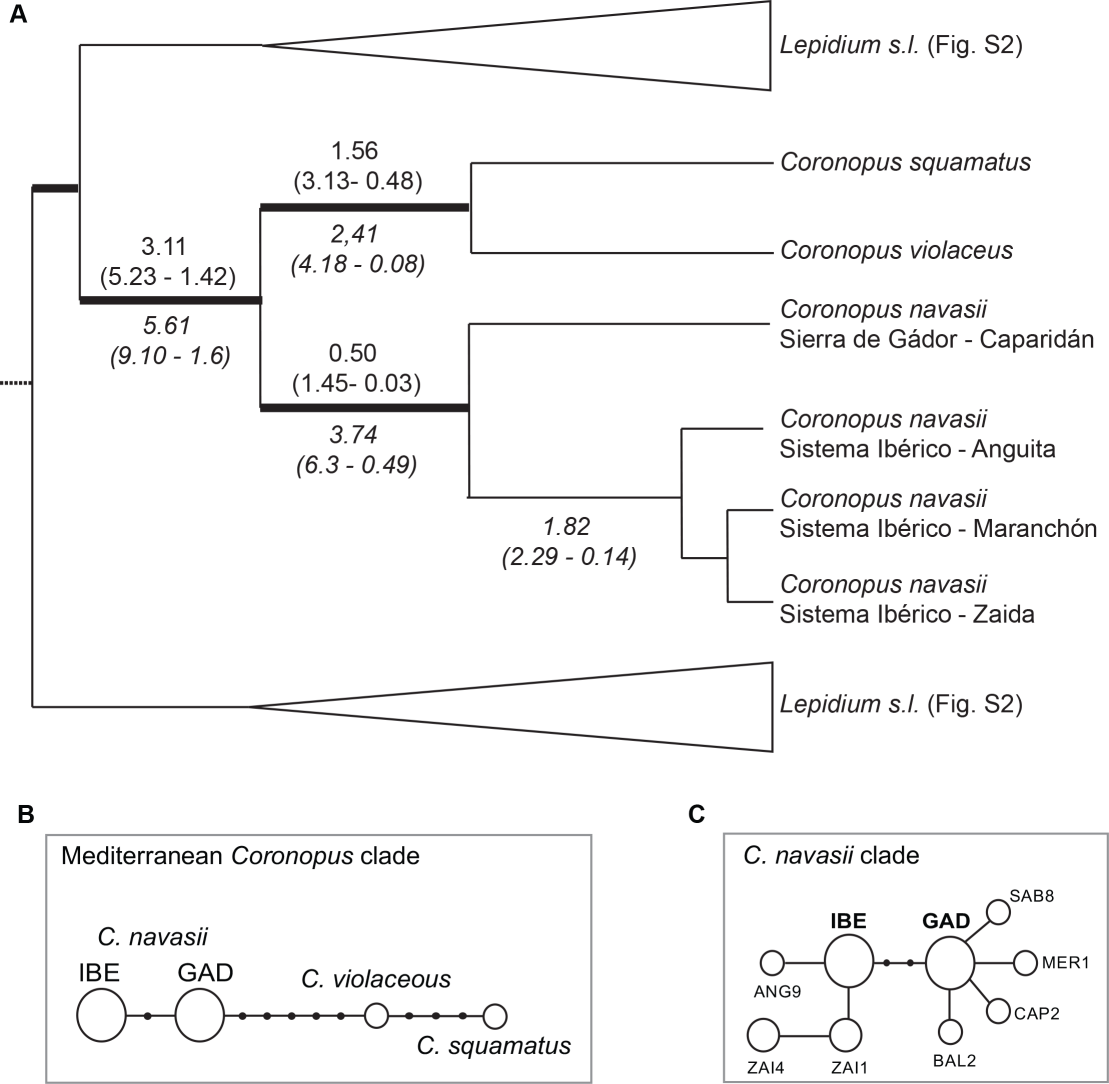
Phylogenetic and phylogeographic results. (a) Phylogenetic reconstruction of the Mediterranean clade of *Coronopus* (*C*. *navasii*, *C*. *squamatus*, *C*. *violaceus*) extracted from the Maximum Clade Credibility trees obtained in BEAST from the nuclear (ITS) and the plastid (*trn*T-*trn*L) *Lepidium* matrices (S4 Fig). Divergence time ages are only specified for supported branches. Thick lines indicate branches with a posterior probability of 1.00 in both the nuclear and plastid analyses. Ages recovered in the analysis of the nuclear *Lepidium* matrix are provided above branches, whereas the ones obtained from the plastid *Lepidium* matrix are indicated below. (b) Haplotype network of the Mediterranean clade of *Coronopus* obtained from the Statistical Parsimony analysis of the plastid *trn*L-*trn*F and *trn*T-*trn*L spacers as implemented in TCS. (c) Haplotype network of the *Coronopus navasii* clade obtained from the Statistical Parsimony analysis of the plastid *trn*T-*trn*L, *rps*16-*trn*Q and *trn*H-*psb*A spacers as implemented in TCS. Correspondence between haplotypes and samples are specified in Table 1.

The topology of the MCC tree obtained from the BEAST analysis of the *Lepidium* nuclear matrix is mostly congruent with previous results based on ITS DNA region [26] displaying identical or higher clade supports (S4A Fig). The MCC tree obtained from the analysis of the *Lepidium* plastid matrix displays a large basal polytomy with few well-supported lineages (S4B Fig). Despite the low resolution of the plastid phylogeny, two incongruences were visually detected when compared to the nuclear phylogeny (S4A vs. S4B Fig). In fact, results from the AU and BF tests reveal significant incongruence between the nuclear and plastid trees topologies (S3 Table), preventing us from conducting a concatenated analysis. Therefore, the results will be discussed independently. *Coronopus navasii* constitutes a well-supported monophyletic species in the MCC tree obtained from *Lepidium* nuclear matrix (1.00 Posterior Probability, PP; Fig 3A and S4A Fig) sister to *C*. *squamatus* and *C*. *violaceus* (hereafter called ‘Mediterranean *Coronopus* clade’; 1.00 PP; Fig 3A and S4A Fig), as already reported by Mummenhoff et al. (2009) [26]. The MCC tree (S4A Fig) recovers 3.11 Mya (95% High Posterior Density Confidence Interval (95% CI): 5.23–1.42 Mya, Fig 3A and S4A Fig) for the early divergence of the Mediterranean *Coronopus* clade. The crown age recovered for *C*. *navasii* is 0.50 Mya (95% CI: 1.45–0.03 Mya; Fig 3A and S4A Fig). The MCC tree obtained from *Lepidium* plastid matrix (S4B Fig) recovers 5.61 Mya (95% CI): 9.10–1.60 Mya, Fig 3A and S4B Fig) for early divergence of the Mediterranean *Coronopus* clade. The divergence between the *Sierra de Gádor* and *Sistema Ibérico* disjunct areas is 3.74 for Mya (95% CI: 6.30–0.49 Mya; Fig 3A and S4B Fig).

#### Genetic variability of *C*. *navasii*

No sequence variation was detected among all individuals of *C*. *navasii* for the two plastid regions *trn*S*-trn*G and *trn*L*-trn*F and the nuclear ITS. The sequence variation of the remaining three plastid regions is as follows: two variable and potentially informative sites in the *trn*T*-trn*L region, two variable and potentially informative sites in *rps*16*-trn*Q and one variable and potentially informative site in *trn*H*-psb*A. All the 24 individuals from *Sierra de Gádor* display identical sequences for the three DNA regions in terms of nucleotide substitutions. Only individual 8 from *Sabinar* pond displays a nucleotide substitution in the *trn*H*-psb*A region. Four 1-pair base indels were detected within *Sierra de Gádor*, each of them exclusive to one individual in each population. The *Sistema Ibérico* populations exhibit more variability than *Sierra de Gádor* in terms of nucleotide substitutions. Six individuals (individuals 1, 2, 4, 6, 7, 10) in the *Zaida* population display one nucleotide substitution in *rps*16*-trn*Q region, and three of these six individuals display a further substitution in *trnH-psbA* region. An additional indel is detected in one individual of the *Anguita* population (individual 9). Individuals from *Sierra de Gádor* differ from those of *Sistema Ibérico* in three nucleotide substitutions.

#### Haplotype networks

The statistical parsimony analysis of the Mediterranean *Coronopus* clade matrix reveals a single substitution-based haplotype network with no loop (Fig 3B). Thirteen haplotypes are recovered, four detected within the dataset and nine missing needed to connect the detected haplotypes. Two geographically well-defined haplotypes are detected within *C*. *navasii* connected through one missing haplotype (Fig 3B). Two haplotypes are detected within the sister-group congruent with the two species included (*C*. *squamatus* and *C*. *violaceous*) and connected through three missing haplotypes. The remaining five missing haplotypes connect the *Gádor* haplotype of *C*. *navasii* to the North African *C*. *violaceous* (Fig 3B).

The statistical parsimony analysis of the *C*. *navasii* matrix recovers a single haplotype network with nine haplotypes found within the *C*. *navasii* dataset and two missing haplotypes (Fig 3C, Table 1). Nineteen of the 24 individuals from *Sierra de Gádor* share the same internal haplotype (GAD) while the remaining four, one from each population, display a different tip haplotype (BAL2, CAP2, MER1, SAB8, Table 1). Twenty individuals of the *Sistema Ibérico* share the same internal haplotype (IBE). Two additional haplotypes are detected in the *Zaida* population (ZAI1, ZAI4), shared by three individuals each and individual 9 from the *Anguita* population displays a different haplotype based on a single-base indel (ANG9). Two missing haplotypes connect the widespread *Sierra de Gádor* haplotype (GAD) with the widespread IBE haplotype from the *Sistema Ibérico* (Fig 3C).

#### Gene flow and genetic differentiation

The AMOVA analysis assigns the greatest amount of variation between the two geographical groups (Central East and Southeast Spain, 84.14%, Table 3). Very limited level of differentiation is detected among populations within groups (5.18%, Table 3) in comparison to the one estimated within populations irrespective to the group of population to which they belong (10.68, Table 3). Results of gene flow estimated with dnaSP are consistent with the degree of genetic isolation inferred from AMOVA results. Gene flow estimation shows a very high genetic differentiation between groups (FST = 0.861) and a low number of migrants per generation (Nm = 0.04). Similarly, GST values are also high (GST = 0.442, Nm = 0.32), also supporting a high level of isolation.

**Table 3:** Genetic differentiation and gene flow estimates recovered from the analyses conducted in Arlequin and dnaSP softwares.

**AMOVA analysis**			
**Source of variation**	**Df**	**Sum of Squares**	**Percentage of variation**
Among groups (Central East–Southeast Spain)	1	38.138	84.14
Among populations within groups	5	3.958	5.18
Within populations	43	8.164	10.68
**Gene flow estimates from DNA sequences**			
FST = 0.861	Number of migrants per generation = 0.04		
**Gene flow estimates from haplotypes**			
GST = 0.442	Number of migrants per generation = 0.32		

### Species distribution modeling

The six applied algorithms perform similarly with only marginal significant differences between models (S4 Table). The minimum mean score obtained is 0.8155 in the ANN model. The presence-absence threshold for the ensemble model maximizes AUC (0.92) at a probability value of 0.55. The predictor importance according to each model is variable between models (Table 2). In general, models allocate more importance to temperature variables or to their interactions with precipitation. For instance, GLM concedes greater importance to annual mean temperature and mean temperature in the driest quarter together with topographic exposure, and much less relevance to hydric constraints. Annual precipitation is in general less relevant.

**Fig 4:**
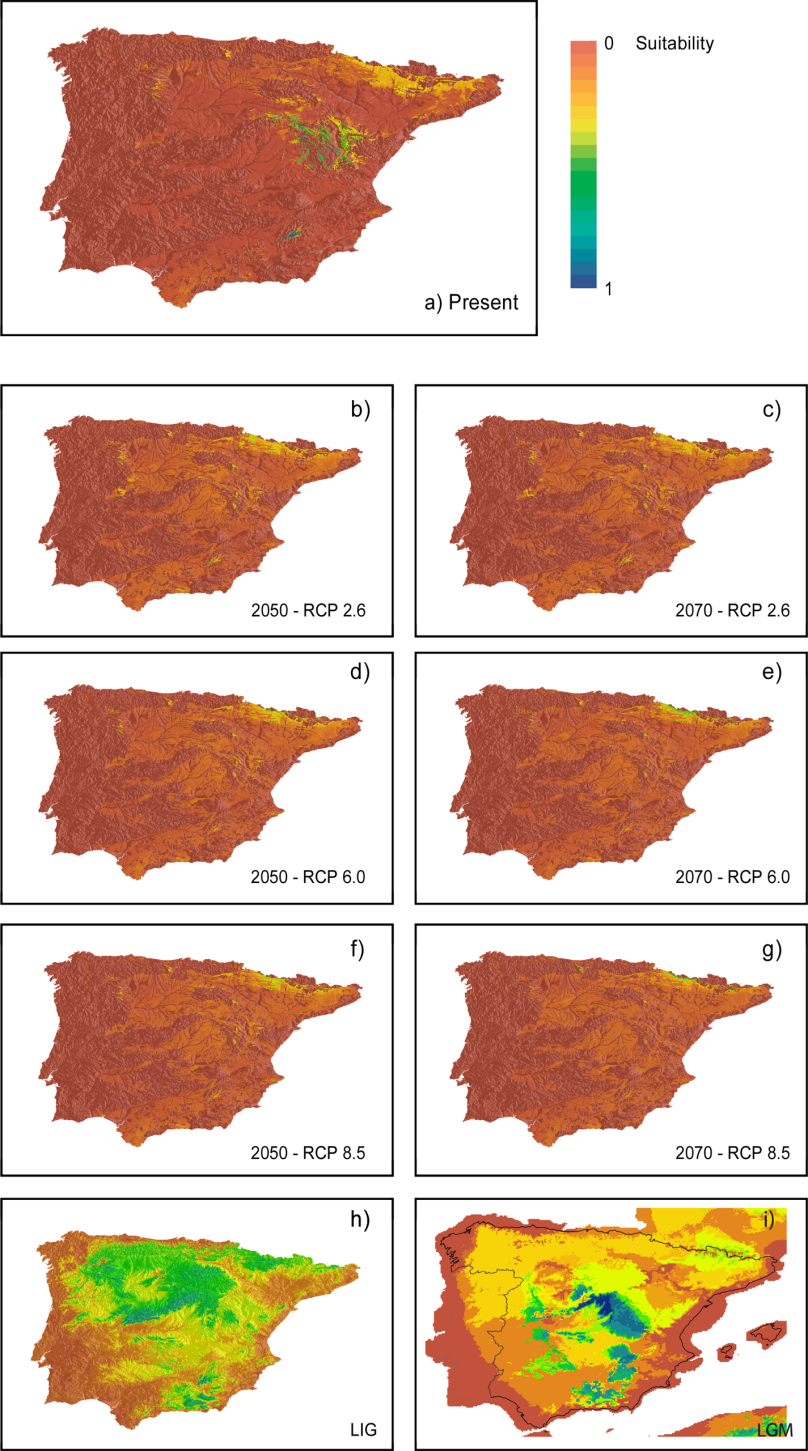
Topoclimatic niche suitability maps. Results from the six algorithms applied to model the species distribution accounting for climate and topographic predictors. A: Present projection masked with clay soils layers; B-G: Future conditions projections under different emissions scenarios masked with clay soils layers; H-I: Climate suitability niche models projected to past conditions to Last Interglacial period (LIG) and Last Glacial Maximum (LGM) period climatic conditions, without considering soil properties.

The current topoclimatic niche suitability predicted by models fits the present-day distribution of the species at high altitudes in *Sierra de Gádor* and *Sistema Ibérico* without filtering distribution with soil properties (S5A Fig). The rest of mountain regions in the Iberian Peninsula show lower suitability. Projected distributions under the three climate change scenarios considering soil properties show very limited niche suitability. The future climate scenarios show a progressive loss of climate suitability in all cases but with differences. Under RCP 2.6 the current mountain ranges where the species is present are maintained, whereas under 6.0 and 8.5 they show a greater decrease. When accounting for clay soils, the decrease of suitable areas is dramatically and the species would eventually disappear from current areas, with only suitable areas remaining in the Pyrenean range. Comparison of lost and gained cells in the present-day model and the projected ones reveals a net decrease in suitability for current areas and a northward increase of suitable areas availability under the three emission trajectories considered (Table 4). This increase is higher for the 2050 period than to the 2070 one, where the expected number of suitable cells decreases in scenarios 2.6 and 8.5. Filtering projected maps with soil properties provides similar results but with a markedly decrease in the availability of suitable areas (Table 4) as demonstrated by suitable maps. Proportion of gained and lost cells differed among RCPs. Scenario 2.6 shows a negative change rate of -4.8%, whereas scenarios 6.0 and 8.5 reveal changes of -24% and +12% respectively (Table 4), the latter due to a range shift towards the Pyrenees. Regarding past climate suitability, the LIG projection shows a wide distribution of available climatic niche including large but discontinuous area between *Sistema Ibérico* and *Sierra de Gádor* (Fig 4H). Conversely, the projection to the LGM period shows less suitable climatic areas in lower altitudes when compared to current predicted potential distribution (Fig 4I).

**Table 4:** Proportion of lost/gained cells between time periods according to three selected scenarios of greenhouse gases EMISSIONS. RCP = Representative Concentration Pathways scenarios of greenhouse gases considered.

RCP scenario	Time period	Percentage of lost cells		Percentage of gained cells		Change rate	
*2*.*6*	*Present- 2050*	63.86	**79.68**	6.471	**108.958**	-57.389	**29.279**
	*Present- 2070*	63.02	**82.156**	6.356	**77.276**	-56.664	**-4.88**
	*2050–2070*	60.769	**93.154**	9.428	**167.444**	-51.341	**74.29**
*6*.*0*	*Present- 2050*	82.54	**98.106**	5.798	**194.61**	-76.742	**96.504**
	*Present- 2070*	60.769	**93.154**	9.428	**167.444**	-51.341	**74.29**
	*2050–2070*	93.195	**99.782**	4.087	**75.601**	-89.108	**-24.181**
*8*.*5*	*Present- 2050*	8.975	**29.183**	10.678	**2.761**	1.702	**-26.423**
	*Present- 2070*	53.797	**59.131**	1.595	**71.876**	-52.202	**12.746**
	*2050–2070*	81.188	**91.809**	3.573	**35.311**	-77.615	**-56.498**

Bold numbers are results accounting for presence of clay soils.

## Discussion

The pre-Holocene context estimated for the divergence between *Sistema Ibérico* and *Sierra de Gádor* allows us rejecting the human-mediated ‘transhumance hypothesis’ for the origin of the *C*. *navasii* disjunction (Fig 3A). A long-term isolation and the need to consider at least two independently evolving (and conservation) units are suggested by (1) the haplotype differentiation between *Sistema Ibérico* and *Sierra de Gádor* without any detected shared haplotype and two missing haplotypes connecting the two disjunct areas (Fig 3B), and (2) the great genetic differentiation and low gene flow suggested by molecular estimates (Table 3) Future climate change models do not predict a decrease of suitable areas but a severe shift in their location (Fig 4B–4G and S5B–S5G Fig) that may require the species migration to survive. This coupled with the rarity of the species habitat and the intrinsic constraints superimposed by the natural fragmentation of this habitat might compromise its long-term survival. These reasons force the imperative implementation of new actions in the *Sistema Ibérico* populations in order to contemplate the whole genetic variability of this species and properly address the above-mentioned challenges faced.

### Pre-Holocene dispersal for *Coronopus navasii* disjunction

The confirmed phylogenetic placement of *C*. *navasii* as sister to the Mediterranean species *C*. *squamatus* and *C*. *violaceus* [26] reflects an interesting biogeographic and environmental congruence (Fig 3A). Both the North African *C*. *violaceous* and the Iberian *C*. *navasii* are hemicryptophytes and restricted endemics to temporary ponds at medium to high altitudes [65]. However, *C*. *violaceus* is more closely related to the annual and widely distributed *C*. *squamatus* than to the alike *C*. *navasii*. *Coronopus squamatus* inhabits ruderal, open and xeric disturbed habitats up to 1,200 m.a.s.l. across the Mediterranean Basin and C European areas [66, 67]. The terminal placement of this widespread species could be interpreted as the acquisition of key innovation features (annuality and low environmental requirements) that may have increased chances of dispersal and population establishment success [66, 67]. The biogeographical scenario under this hypothesis implies a geographically confined ancestor for the Mediterranean clade of *Coronopus* with the range expansion occurring along the branch of *C*. *squamatus*. An alternative but less plausible explanation is that annuality and low environmental requirements were ancestral in the clade, being hemicryptophyte habit and high habitat specificity acquired independently both in the Iberian Peninsula (*C*. *navasii*) and North Africa (*C*. *violaceus*). This evolutionary path would resemble the general tendency observed among the California flora, where endemic specialists of 'vernal pools' are derived from 'terrestrial antecessors', although in these cases acquisition of annuality is linked to the ‘vernal pool’ specialists [68, 69]. Under this hypothesis a widespread ancestor of the Mediterranean clade of *Coronopus* would be expected. To address these questions with certainty, a comprehensive sample embracing the whole *C*. *squamatus* distribution and the inclusion of the fourth Mediterranean species of *Coronopus* (*C*. *lepidioides* (Coss. & Durieu) Kuntze, North Africa) are needed together with a comparative study of the phylogenetic signal of traits related to dispersal capacity.

The Miocene-Pleistocene origin for the *C*. *navasii* internal divergence (6.30–0.49 Mya according to *trn*T-*trn*L and 1.45–0.03 Mya according to ITS; Fig 3A) does not fit the Holocene divergence (< 0.01 Mya) that would be expected under the ‘transhumance hypothesis’, even considering the great uncertainty reflected by the large Confidence Intervals detected. In fact, this traditional land use became an active practice in Spain mainly in historic times and reached its peak during the first quarter of XVI Century [23]. Therefore, transhumance can be ruled out as a plausible explanation for the origin of the *C*. *navasii* disjunction (Fig 3A). On the other hand, vicariance does not emerge as an alternative hypothesis for reasons added to the natural fragmentation of *C*. *navasii* habitat. Also, the lack of any shared haplotype as a vicariance hypothesis may predict and the already scattered suitable areas distribution in the past inferred in this study are not congruent with a vicariance scenario.

The most plausible scenario for the *C*. *navasii* disjunction is a northward dispersal from *Sierra de Gádor* to the *Sistema Ibérico* as inferred by the fact that the *C*. *navasii* network is connected to the sister-group through a haplotype from *Sierra de Gádor* (Fig 3B). This northward direction is consistent with the northward progressive climatic recovery identified after the LGM from paleobotanical and geological data [70] that has also been used to explain other mountain plant disjunctions in the Mediterranean [71, 72]. Also, a similar pathway of northward dispersal has been proposed for Iberian xerophytes such as *Ferula loscosii* (Apiaceae) or the *Vella pseudocytisus-V*. *aspera* complex during the Pliocene [73, 74] as the onset of the Mediterranean climate was creating dry continental niches in NE Iberia. From a biological point of view, dispersal is plausible since epizoochory has been described as the secondary dispersal mechanism for *C*. *navasii* [5]. Besides, *C*. *navasii* fruit phenology coincides with summer movements of migrating animals that use these ponds as water supplies ([16, 75], Fig 2), which are particularly essential in semiarid environments like the ones where *C*. *navasii* occurs [76]. However, the *Sierra de Gádor*—*Sistema Ibérico* path is not a common migratory route for birds, at least currently [28]. Finally, whether the dispersal was the result of long distance dispersal (LDD) or stepping-stone (SSD) process remains open. The appearance of intermediate suitable areas in past projections (Fig 4H–4I) makes the SSD hypothesis likely, although LDD cannot be ruled out.

### Conservation implications and management strategy

Our findings on the haplotype differentiation between the *Sistema Ibérico* and *Sierra de Gádor* urge the implementation of targeted local actions into the *Sistema Ibérico* populations in a coordinated action with the on-going *Sierra de Gádor* program. We suggest considering both areas as independent operational conservation units (OCUs). The > 500 km distance between both areas limits the implementation of common legal actions because of different regional political competences. Moreover, the conservation strategy of the *Sierra de Gádor* metapopulation does not seem enough to preserve the total genetic diversity of the species due to the different haplotypes and great isolation detected in the *Sistema Ibérico*. The recognition of at least two OCUs is therefore not only a functional proposal, but also an evolutionary-oriented action.

A set of *ex situ* actions must be taken on the two new populations found in 2014 including collecting seeds to preserve in germplasm banks. In parallel, a proper evaluation of the inter-annual demographic fluctuation of each of the three *Sistema Ibérico* populations through consecutive annual censuses is needed. The four censuses available from the *Anguita* population reveal significant oscillations in population size (<100 reproductive individuals in 2004 [16]; ca. 800 in 2012 [28]; ca. 600 in 2014 and ca. 1000 in 2015). However, the limited number of censuses together with the eight years lasting between the first two makes any inference rather speculative. A targeted and effective sampling of suitable areas based on our niche modeling results is recommended, given the occurrence of similar ponds in other places of the *Sistema Ibérico* and surrounding areas.

Temporary ponds are waterholes subjected to extremely changing seasonal environmental conditions that are particularly stressed in the area of study because of the inter-annual rainfall fluctuation superimposed by the unpredictable Mediterranean climate. The general importance of environmental variables that niche models attribute to temperature, precipitation and topography are consistent with this interpretation. Temperature variables scored higher than precipitation and topography across the majority of the models, suggesting that altitude, which determines thermic conditions, can be a strong limiting factor for the species distribution. The lower scores assigned to precipitation variables may be explained indeed by the mentioned higher fluctuation in rainfall regimes, which lowers the explanatory ability of these constraints. Nevertheless, additional analyses at finer scales when spatial data are available would be required to clarify the actual role of predictors. The application of a finer resolution is especially relevant when local topography is an important constraint such as it is in the temporary ponds of *C*. *navasii* (e.g. [77]). Accounting for spatial autocorrelation would be important too because of the aggregated distribution of the individuals of *C*. *navasii* around the ponds. Our results on future projections confirm climate change as a likely driving force promoting extinction in *C*. *navasii* by: (1) accelerating local extinction and migration to higher altitudes or latitudes, and (2) altering the seasonal water system of the ponds where the species occurs due to the predicted aridity and evapo-transpiration rates increase. Therefore, linking inter-annual climate variability to demographic fluctuations might be critical to propose effective actions to face climate change, since future projections predict northward shift in the species distribution coupled with a decrease in habitat suitability in current sites (Fig 4B–4G).

In addition to the demographic and genetic consequences derived from the annual and inter-annual environmental variation, *C*. *navasii* is constrained by the limited patch size and habitat fragmentation that naturally characterized its habitat [78]. The higher levels of genetic variation detected within populations (11%) than among populations (5%) is interpreted as evidence that both disjunct areas follow a metapopulation dynamics (Table 3). Description of the metapopulation dynamic in the *Sistema Ibérico* is key to determine the impact of local inter-annual demographic fluctuations to the overall system. Indeed, livestock have already been suggested as key factors to keep the metapopulation dynamics in *Sierra de Gádor* (Fig 2; [5]). Determining the actual impact of flock movements in *C*. *navasii* dispersal between local populations and new pond colonization rates would be interesting to design a sustainable management of flock activity that ensures the long-term survival in both disjunct areas.

Mediterranean temporary ponds are isolated and uncommon habitats essential for migratory animals [75] that hold a high variety of rare endemic species. Only a few local studies on the flora and vegetation of these ponds [21, 79] have been conducted to improve knowledge and conservation information on plant biodiversity in these microhabitats [78, 80]. This sharply contrasts with the large number of studies performed in other parts of the world on the flora of seasonal ponds in Mediterranean climates (Chile, Australia, California; [68]). Because of limited water availability in the Mediterranean climate, humans have traditionally used these ponds for agriculture, flock watering or domestic supply. The species living in these ponds and all the associated interaction networks depend on the hydrological annual cycle maintenance. However, during last decades, drainage for extensive agriculture or non-controlled livestock watering is leading to a premature drying out of these ponds [81]. Artificial flooding of ponds during summer is also a common practice that has been reported as a negative activity for the community survival [19, 82]. Considering individuals of *C*. *navasii* produce their aerial parts as water drains, the maintenance of the annual water dynamics of the pond is essential. Artificial flooding leading to a permanent water level in ponds is therefore a harmful practice for *C*. *navasii* survival. However, controlled artificial flooding that ensures maintenance of the natural dynamics of water ponds would be a positive management practice since it will ensure the preservation of the species.

## Supporting Information

S1 Fig *Coronopus navasii* growing in the mud. A: detail of a reproductive individual in bloom (*Sierra de Gádor*); B: detail of the root and basal rosette (*Sistema Ibérico*).
(TIF)

S2 Fig Maximum Clade Credibility tree obtained from the Bayesian analysis implemented in BEAST of the *matK* plastid gene from 79 Malvidae sequences using *Gerrardina* (Huertales) and *Tapiscia* (Crossosomatales) as outgroup. Green circles highlight nodes selected as calibration points for the *Lepidum* divergence age estimation. Nodes used as calibration points (red asterisks) in each analysis are numbered according to the text.
(PDF)

S3 Fig Histogram of the sum of squared deviation for each of the four fossil nodes when used as single calibration point. (PDF)

S4 Fig Maximum Clade Credibility tree obtained from the Bayesian analysis implemented in BEAST. Nodes used as calibration points (red asterisks) in each analysis are numbered according to the text. (a) Chronogram from the *nr*DNA ITS region of 106 samples of *Lepidium s*.*l*. plus *Hornungia petraea* as the outgroup. (b) Chronogram from the plastid *trn*T-*trn*L spacer of 106 samples of *Lepidium s*.*l*. plus *Hornungia petraea* as the outgroup.
(PDF)

S5 Fig Niche suitability maps resulting from the sixth algorithms applied to model the species distribution accounting only for climate and topographic predictors but not masked with clay soils layers. (a) Present projections. (f-g) Future conditions under different emissions scenarios.
(PDF)

S1 Table List of the studied material of *Lepidium s*.*l*. used for the phylogenetic-based analyses. (DOCX)

S2 Table List of the studied material of Malvidae used for the divergence age estimate. (DOCX)

S3 Table Results from the Bayes Factor (BF) and Approximate Unbiased (AU) hypothesis test analyses. Bayes Factors for the competing hypotheses are provided. Difference in the Likelihood between the competing hypotheses and the best topology as well as the p-values obtained from the AU test are given. Asterisks indicate evidence in favor to that hypothesis for the BFs and not rejected hypotheses in the AU tests.
(DOCX)

S4 Table Mean ± SD of AUC scores for the six algorithms used in distribution models. (DOCX)
