# Correction: Pre-Holocene Origin for the *Coronopus navasii* Disjunction: Conservation Implications from Its Long Isolation

**DOI:** 10.1371/journal.pone.0173840

**Published:** 2017-03-08

**Authors:** Sara Martín-Hernanz, Alejandro G. Fernández de Castro, Juan Carlos Moreno-Saiz, Virginia Valcárcel

There is an error in the Materials and Methods section. Within the Field and lab work strategy subsection, information regarding two samples from the study is incorrect. The second sentence after Table 1 should read: Individual 1 and individual 2 from la Zaida were collected by S. López and C. Fabregat (20-08-2014) and the samples are herbarium specimens obtained from the Jardí Botànic de la Universitat de València (VAL-221325).
