# Supplementary figures and images for: Pre-Holocene Origin for the Coronopus navasii Disjunction: Conservation Implications from Its Long Isolation
Source: PLoS One. 2016 Jul 27;11(7):e0159484. doi: 10.1371/journal.pone.0159484 (PMC4963129; doi:10.1371/journal.pone.0159484)

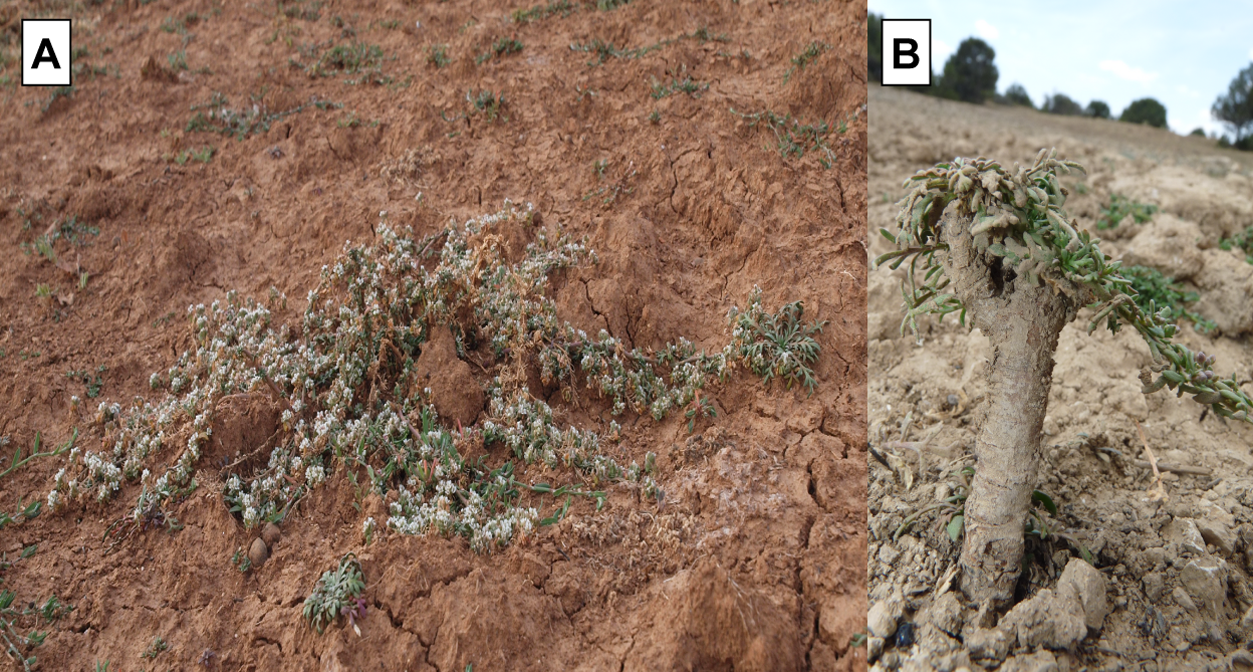

Supplement: S1 Fig — A: detail of a reproductive individual in bloom (Sierra de Gádor); B: detail of the root and basal rosette (Sistema Ibérico). (TIF) [file pone.0159484.s001.tif]

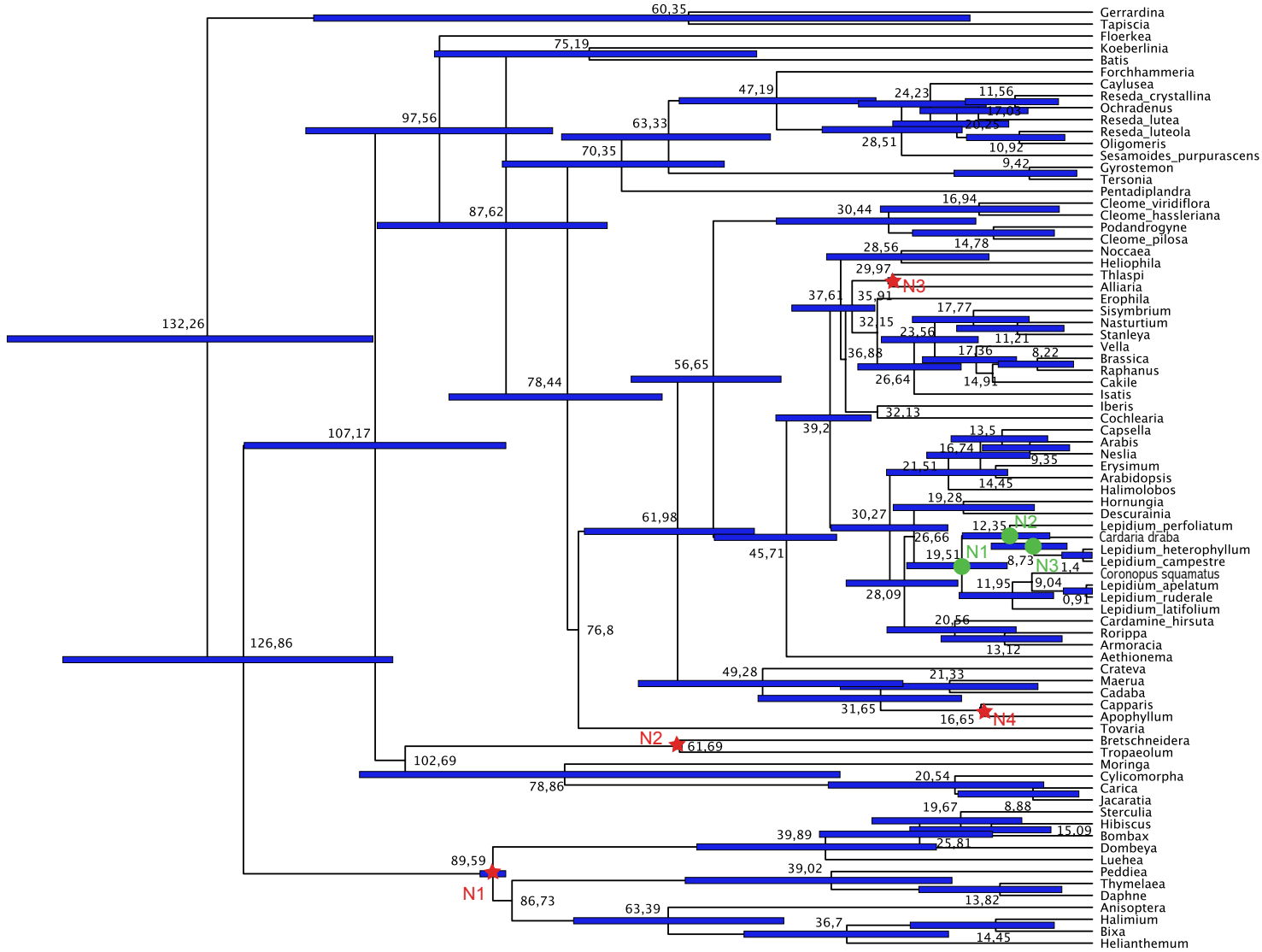

Supplement: S2 Fig — Green circles highlight nodes selected as calibration points for the Lepidum divergence age estimation. Nodes used as calibration points (red asterisks) in each analysis are numbered according to the text. (PDF) [file pone.0159484.s002.pdf]

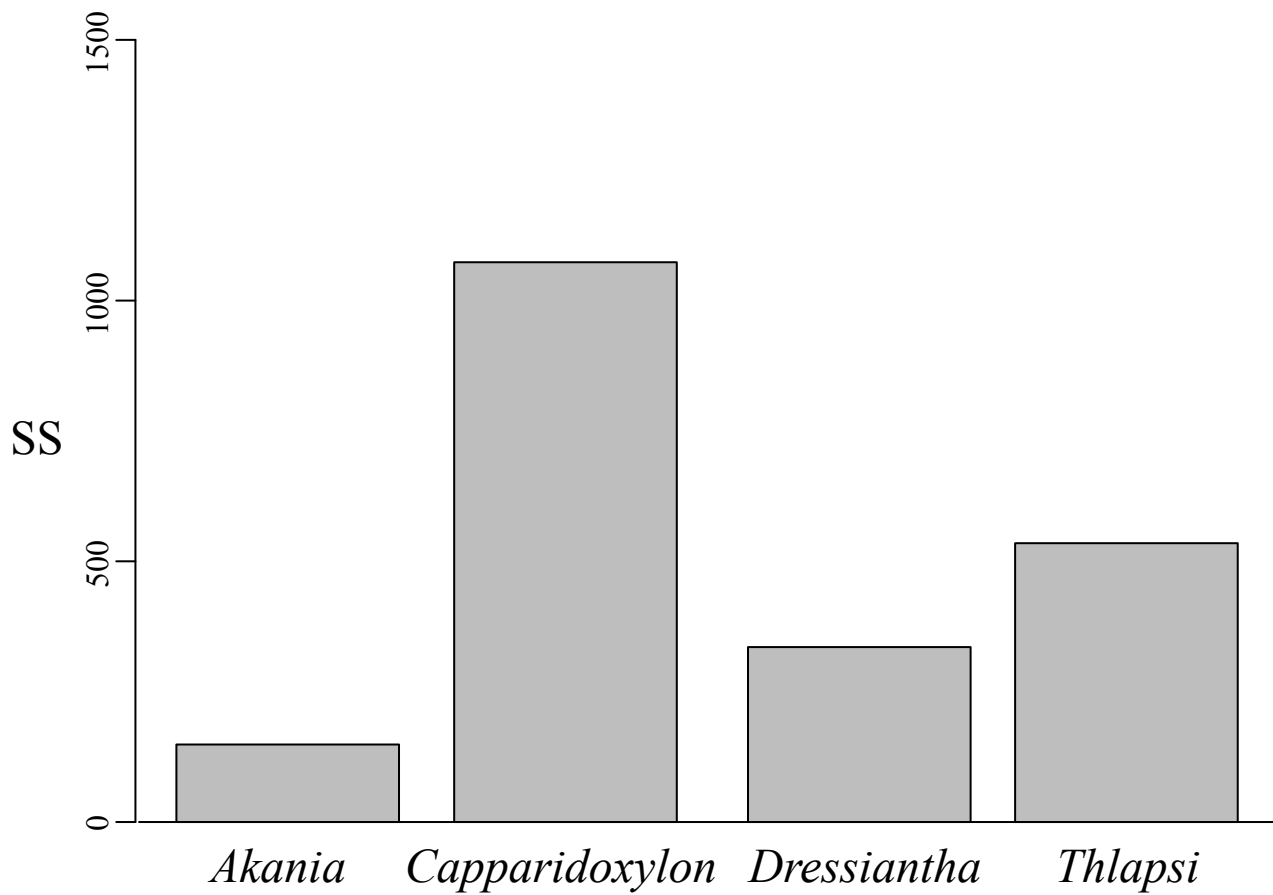

Supplement: S3 Fig — (PDF) [file pone.0159484.s003.pdf]

A

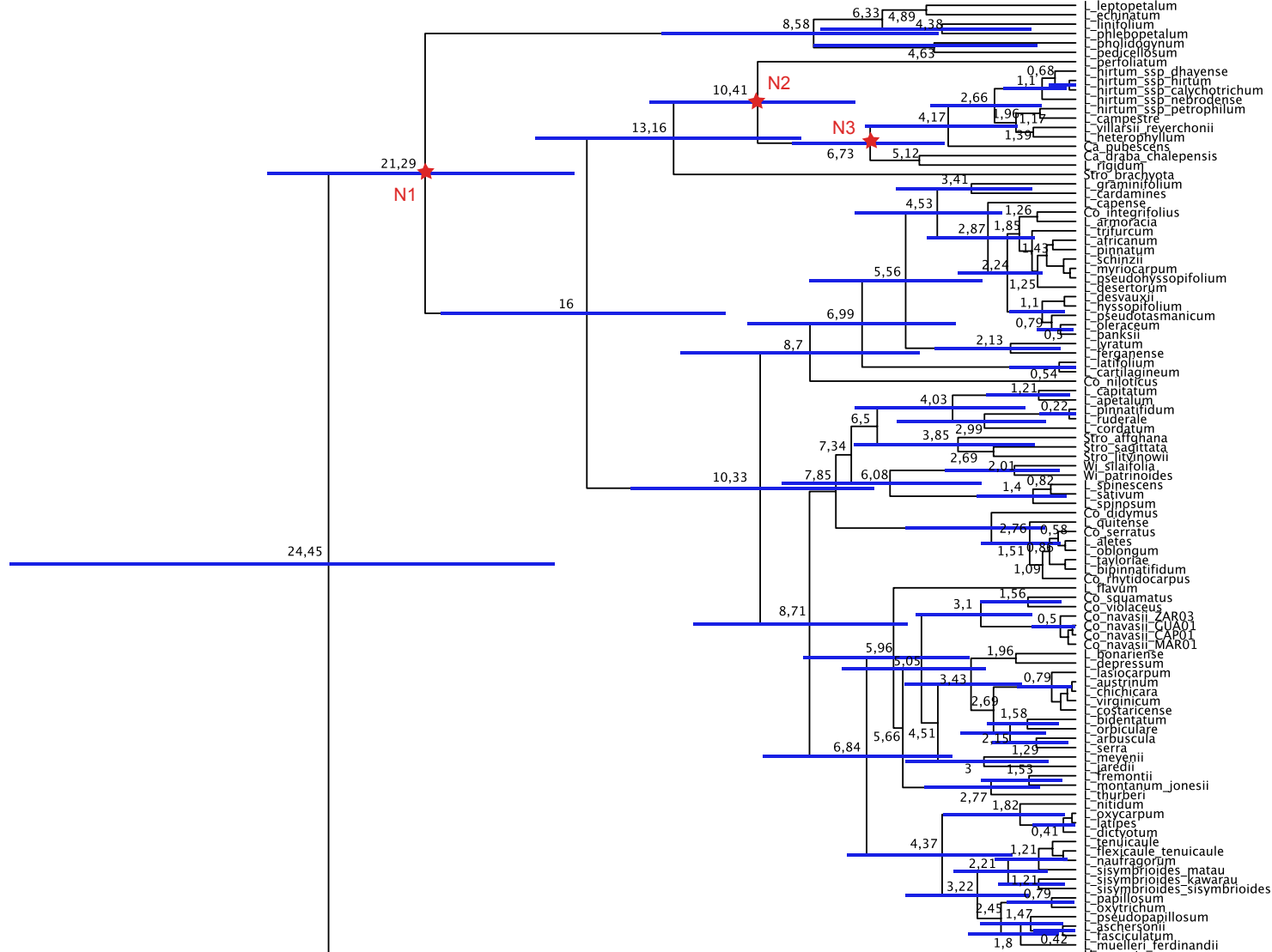

B

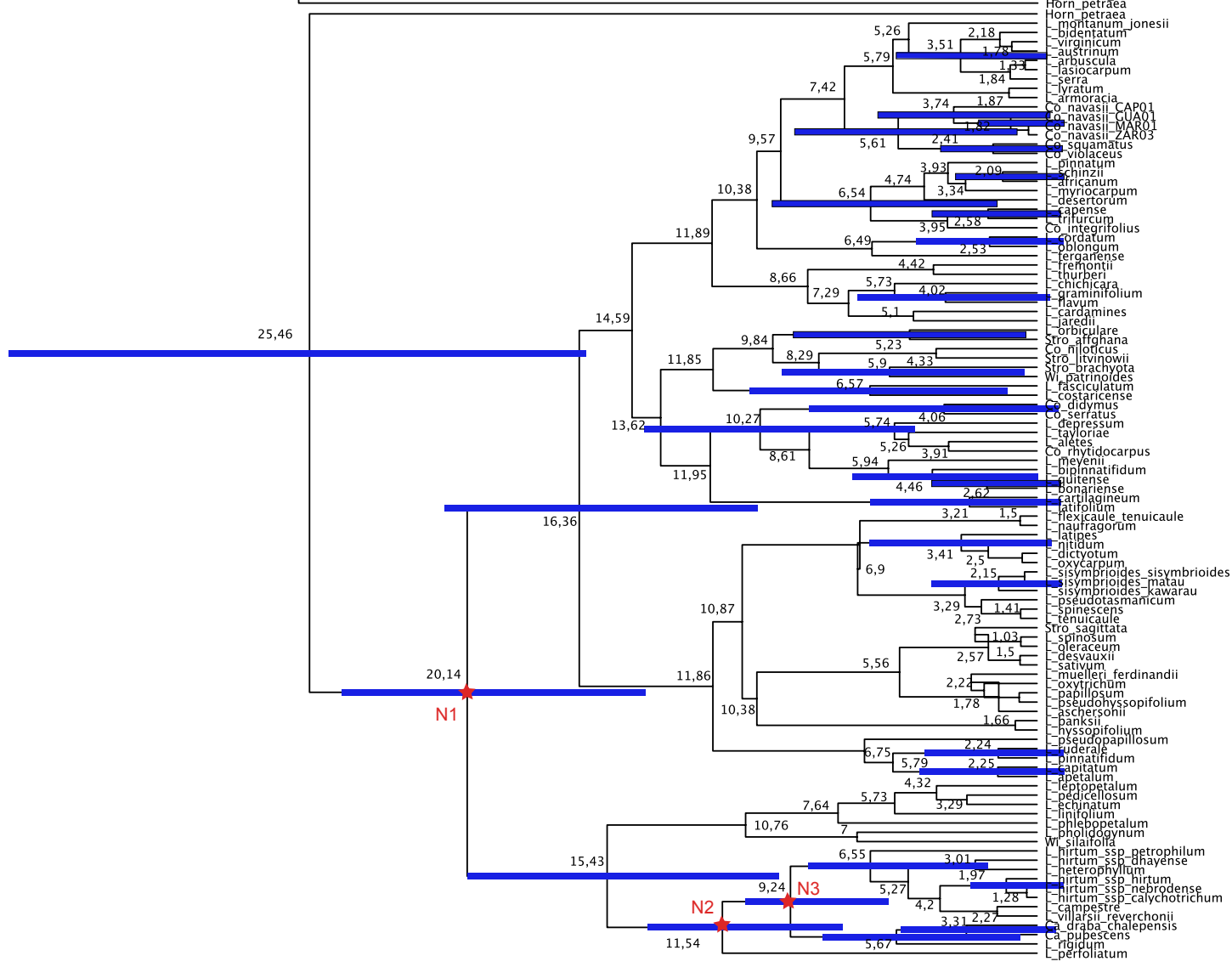

Supplement: S4 Fig — Nodes used as calibration points (red asterisks) in each analysis are numbered according to the text. (a) Chronogram from the nrDNA ITS region of 106 samples of Lepidium s.l. plus Hornungia petraea as the outgroup. (b) Chronogram from the plastid trnT-trnL spacer of 106 samples of Lepidium s.l. plus Hornungia petraea as the outgroup. (PDF) [file pone.0159484.s004.pdf]

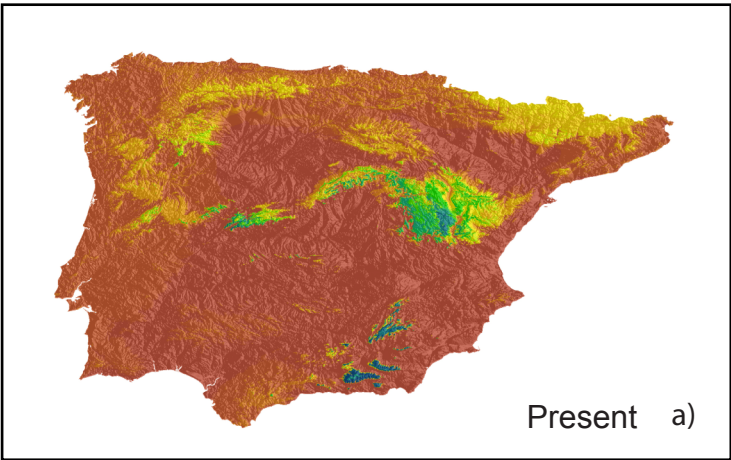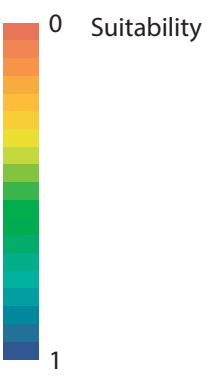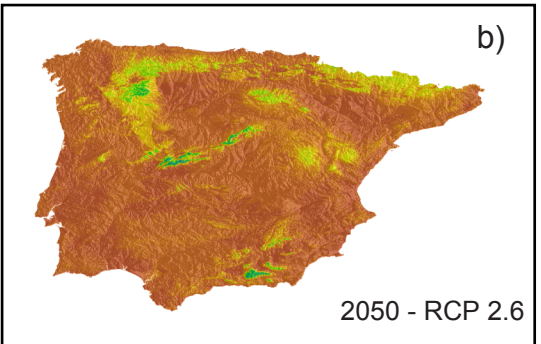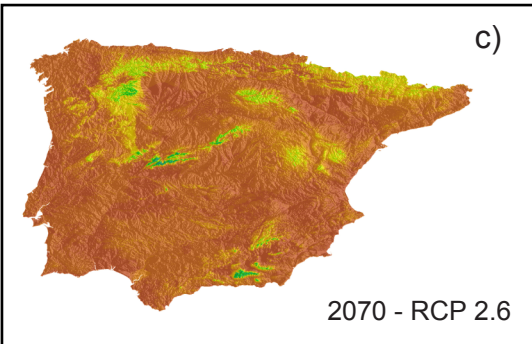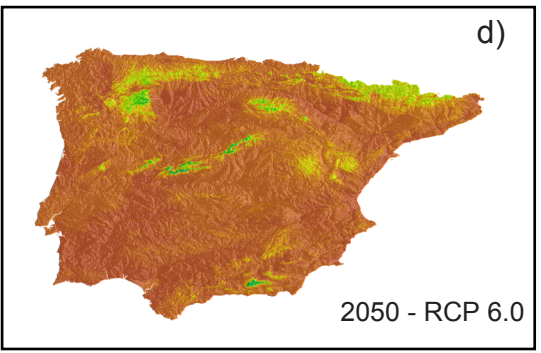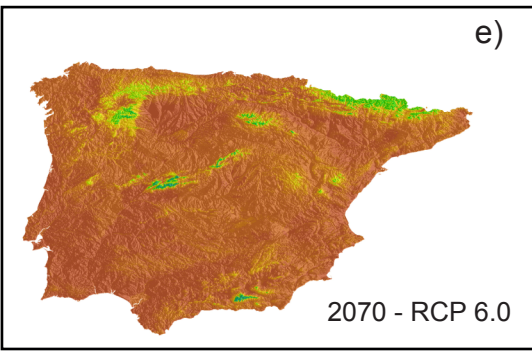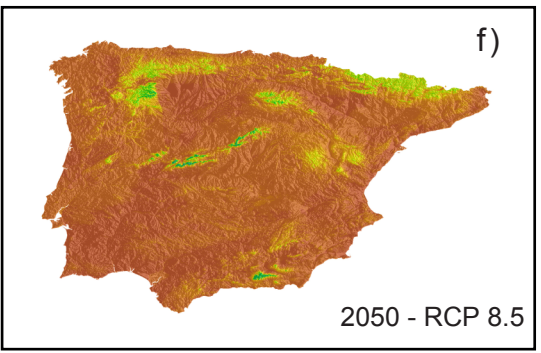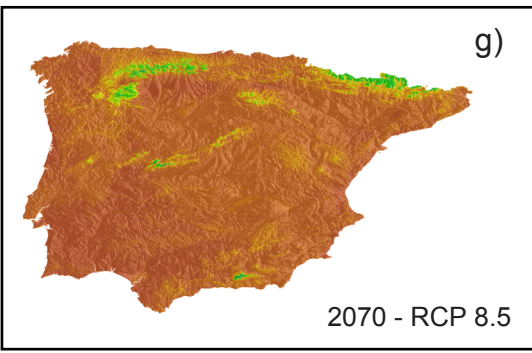

Supplement: S5 Fig — (a) Present projections. (f-g) Future conditions under different emissions scenarios. (PDF) [file pone.0159484.s005.pdf]
