# Supplementary material for: Pre-Holocene Origin for the Coronopus navasii Disjunction: Conservation Implications from Its Long Isolation
Source: PLoS One. 2016 Jul 27;11(7):e0159484. doi: 10.1371/journal.pone.0159484 (PMC4963129; doi:10.1371/journal.pone.0159484)
Supplement: S1 Table — (DOCX) [file pone.0159484.s006.docx]

**S1 Table. List of the studied material of *Lepidium s.l.* used for the phylogenetic-based analyses.**

| Taxón | Origin / Source / Collector | GenBank accession number | | | |
| --- | --- | --- | --- | --- | --- |
|  |  | ITS1 | ITS2 | *trn*T-*trn*L spacer | *trn*L-*trn*F spacer |
| *L. africanum* (Burm.f.) DC.^1^ | South Africa, Cape Province, Williston District / PRE, 95056-102 / G. Germizhuizen | AJ582441 | AJ582498 | AY015703, AY015704 | _ |
| *L. apetalum* Willd.^1^ | China, Beijing / B.G. Beijing, China / s.n. | AJ582466 | AJ582514 | AY015823 | _ |
| *L. aletes* J.F.Macbr.^2^ | Paraguay, Guaira, Cordillera de Ybytyruzú / MO 3927367 / E. Zardini | FM178548 | FM178549 | AM991817 | _ |
| *L. arbuscula* Hillebr.^1^ | Hawaii, Oahu , Makua Valley, Ohikilolo Ridge / National Tropical BG Lawai, Kauaii, Hawaii, 945176 / S. P. Pearlman | AJ582451 | AJ582517 | AY015707, AY015708 | _ |
| *L. armoracia* Fisch. et Mey.^1^ | Arabian Republic Yemen, Shibam / K, MWC 2307 / A. G. Miller 408 | AJ582454 | AJ582502 | AY015709, AY015710 | _ |
| *L. aschersonii* Thell.^1^ | Australia, Victoria, Lake Omeo / La Trobe Univ., Australia, s.n. / N. H. Scarlett | AJ582426 | AJ582483 | AY015711, AY015712 | _ |
| *L. austrinum* Small^1^ | USA, Texas, Hidalgo Co. / DAV Tucker Herbarium, Univ. of California, Davis, 17451 / L. H. Shinners | AJ582467 | AJ582515 | AY015715, AY015716 | _ |
| *L. banksii* Kirk^1^ | New Zealand, Abel Tasman National Park, Totaranui / Dept. of Conservation, Auckland Conservancy, 942179 / P. de Lange | AJ582433 | AJ582490 | AY015717, AY015718 | _ |
| *L. bidentatum* Montin^1^ | Hawaii, Kauaii, Haupu / National Tropical BG Lawai, Kauaii, Hawaii, 925054 / S. P. Pearlman | AJ582468 | AJ582516 | AY015719, AY015720 | _ |
| *L. bipinnatifidum* Desv.^1^ | Bolivia, La Paz, railway station / Osnabrück Univ., Germany / K. Mummenhoff & H. Brüggemann 451 | AJ582446 | AJ582522 | AY015721, AY015722 | _ |
| *L. bonariense* L.^1^ | Chile, Prov. Atacama, Dept. Valle Iorquera / CETYBO, 3078 / O. Zöllner | AJ582458 | AJ582506 | AY015723, AY015724 | _ |
| *L. campestre* (L.) R. Br.^1^ | France, Meurthe-et-Moselle, Villers-les-Nancy / BG Nancy, France / s.n. | AJ582412 | AJ582469 | AY015725, AY015726 | _ |
| *L. capense* Thunb.^1^ | South Africa, Cape Province / PRE, 95056/49 / H. C. Taylor | AJ582452 | AJ582500 | AY015727, AY015728 | _ |
| *L. capitatum* J.D. Hooker & Thomson^2^ | China, Qinghai, Yushu Xian / MO, 04920182 / D.E. Boufford et al. | FM178552 | FM178553 | AM991818 | _ |
| *L. cardamine* L.^2^ | Spain / INIA Madrid, Index 1990, 421-1572-68 / s.n. | FM178554 | FM178555 | AM991819 | _ |
| *L. cartilagineum* (J.Meyer) Thell.^2^ | Germany, Thuringia / BG Univ. Jena, Index 1994/95, No. 1916 / s.n. | AM991703 | AM991704 | AM991820 | _ |
| *L. chichicara* Desv.^2^ | Bolivia, Depto. La Paz, Prov. Murillo, La Paz - Cota Cota / MO, 04594519 / St. G. Beck 11148 | AM991705 | AM991706 | AM991821 | _ |
| *L. cordatum* Willd. ex DC.^2^ | Mongolia / BG Kopenhagen, Index No. 4170 / s.n. | AM991707 | AM991708 | AM991822 | _ |
| *L. costaricense* Thell.^2^ | Costa Rica, Monteverde / Univ. Osnabrück, Germany / K. Mummenhoff 446 | AM991711 | AM991712 | AM991825 | _ |
| *L. depressum* Thell.^2^ | Bolivia, Depto. La Paz / MO, 3197061 / J.C. Solomon 11457 | AM991717 | AM991718 | AM991826 | _ |
| *L. desertorum* Eckl. & Zeyh.^1^ | South Africa, Cape Province / PRE, 95056/68 / M B. Bayer | AJ582453 | AJ582501 | AY015729, AY015730 | _ |
| *L. desvauxii* Thell.^1^ | Australia, Victoria, Townsend / La Trobe Univ., Australia / N. H. Scarlett s.n. | AJ582429 | AJ582486 | AY015731, AY015732 | _ |
| *L. dictyotum* A. Gray^1^ | USA, California, San Luis Obispo Co. / Tucker Herbarium, Univ. of California, Davis, 32057 / E. C. Twisselmann | AJ582415 | AJ582472 | AY015733, AY015734 | _ |
| *L. echinatum* Hewson^2^ | Australia / Bentley Delivery Centre, Australia / s.n. | AM991719 | AM991720 | AY015735, AY015736 | _ |
| *L. fasciculatum* Thell.^1^ | Australia, Victoria, river road near Lake Walla Walla / La Trobe Univ., Australia, s.n. / J. H. Browne | AJ582428 | AJ582485 | AJ582562 | _ |
| *L. ferganense* Korsh.^1^ | Russia, Moscow / BG Moscow, Russia / s.n. | AJ582449 | AJ582519 | AY015737, AY015738 | _ |
| *L. flavum* Torr.^1^ | USA, Nevada, Churchill Co., Slate Mountain / MO / A. Tiehm & P. Lott, 4011 | AJ582444 | AJ582524 | AY015739, AY015740 | _ |
| *L. flexicaule* Kirk^1^ | New Zealand / Auckland BG, cultivated plants, 950769 / s.n. | AJ582430 | AJ582487 | AY015741, AY015742 | _ |
| *L. fremontii* S. Wats.^1^ | USA, California, Joshua Tree Desert / Univ. Osnabrück, Germany / H. Hurka, s.n. | AJ582456 | AJ582504 | AY015815 | _ |
| *L. graminifolium* L.^2^ | Germany, Göttingen / OSBU 90-08-0096-50 / s.n. | AM991721 | AM991722 | AM991823 | _ |
| *L. heterophyllum* (DC.) Benth.^2^ | Germany, Lauferbacherhof/Ahr / Univ. Osnabrück, Germany / s.n. | AM991693 | AM991694 | AY015816 | _ |
| *L. hirtum* (L.) Sm. ssp. *calycotrichum* (Kunze) Thell.^2^ | Spain, North Almeria / ETSIA, 427-0926-66 / s.n. | AM991695 | AM991696 | AY015817 | _ |
| *L. hirtum* (L.) Sm. ssp. *dhayense* (Manby) Thell.^2^ | Morocco, near Oukaimeden / ETSIA, 428-2178-72 / s.n. | AM991697 | AM991698 | AY015818 | _ |
| *L. hirtum* (L.) Sm. ssp. *hirtum*^2^ | France, Dept. Aude, Montagne de Tauch / BG Univ. Liege, Belgium, 85-3863 / s.n. | AJ582413 | AJ582470 | AY015819 | _ |
| *L. hirtum* (L.) Sm. ssp. *nebrodense* (Rafin) Thellung^2^ | Italy, Sizilia / ETSIA, 429-6201-83 / s.n. | AM991699 | AM991700 | AY015820 | _ |
| *L. hirtum* (L.) Sm. ssp. *petrophilum* (Cosson) Thell.^2^ | Spain, Sierra Nevada / BG Marburg, Germany, 83-80 / s.n. | AM991701 | AM991702 | AY015821 | _ |
| *L. hyssopifolium* Desv^1^ | Australia, Victoria, Beveridge / La Trobe Univ., Australia, 70-296-940 / N. H. Scarlett | AJ582435 | AJ582492 | AY015743, AY015744 | _ |
| *L. jaredii* Brandegee^2^ | USA, California / Univ. of California, Davis 131808 / P. Shiffman | AM991733 | AM991734 | AM991824 | _ |
| *L. lasiocarpum* Nutt.^1^ | USA, California, Joshua Tree Desert / ETSIA, 430-1738-69 / s.n. | AJ582455 | AJ582503 | AY015745, AY015746 | _ |
| *L. latifolium* L.^1^ | Germany, Leipzig, garbage dump / BG Leipzig, Germany / P. Gutte | AJ582447 | AJ582521 | AY015747, AY015748 | _ |
| *L. latipes* Hook.^1^ | USA, California, Solano Co. / Tucker Herbarium, Univ. of California, Davis, 37209 / J. M. Tucker | AJ582416 | AJ582473 | AY015749, AY015750 | _ |
| *L. leptopetalum* F. Muell.^2^ | Australia, Viktoria / La Trobe Univ., Australia / s.n. | AM991737 | AM991738 | AY015751, AY015752 | _ |
| *L. linifolium* (Desv.) Steud.^2^ | Australien, Carnavon / Kings Park & BG, Perth, Australia / s.n. | AM991739 | AM991740 | AY015753, AY015754 | _ |
| *L. lyratum* L.^1^ | Iran, mountains near Abadeh / ETSIA, 433-3758-75 / s.n. | AJ582448 | AJ582520 | AY015755, AY015756 | _ |
| *L. meyenii* Walpers^1^ | Peru, Dept. Junin, Huayre / M. Hermann, Intern. Potato Center, Quito, Ecuador, JTA-106 / J. Anco | AJ582445 | AJ582523 | AY015757, AY015758 | _ |
| *L. montanum* Nutt.^1^ | USA, Arizona, Shonto / BG Univ. Liege, Belgium, s.n. / s.n. | AJ582457 | AJ582505 | AY015759, AY015760 | _ |
| *L. muelleri-ferdinandi* Thell.^1^ | Australia, New South Wales , Menindee Lakes / La Trobe Univ., Australia, s.n. / J. H. Browne | AJ582427 | AJ582484 | AY015761, AY015762 | _ |
| *L. myriocarpum* Sond.^1^ | South Africa, Cape Province, Vaalbos National Park / PRE, 95056/53 / P. C. Zietsman | AJ582442 | AJ582499 | AY015763, AY015764 | _ |
| *L. naufragorum* Garnock-Jones & D.A. Norton^1^ | New Zealand / P. de Lange, cultivated plants, Dept. Conserv., Auckland Conservancy, New Zealand, 950771 / s.n. | AJ582422 | AJ582479 | AY015765, AY015766 | _ |
| *L. nitidum* Nutt.^1^ | USA, California , Table Mountains / Univ. Osnabrück, Germany / H. Hurka, 338 | AJ582414 | AJ582471 | AY015767, AY015768 | _ |
| *L. oblongum* Small^1^ | Cultivated plants / BG Copenhagen, Denmark / s.n. | AJ582462 | AJ582510 | AY015769, AY015770 | _ |
| *L. oleraceum* Sparrm.^1^ | New Zealand, Port Waikato, Ngatutura Point / Dept. Conserv., Auckland Conservancy, New Zealand, 941265 / P. de Lange | AJ582434 | AJ582491 | AY015771, AY015772 | _ |
| *L. orbiculare* St. John^2^ | USA, Kanai / Smithsonian Institution Washington D.C. / Wood & Perlman 2814US | AM991743 | AM991744 | AM991828 | _ |
| *L. oxycarpum* Torrey & A.Gray^1^ | USA, California, Merced Co. / Tucker Herbarium, Univ. of California, Davis, 115743 / C. A. & L. P. Janeway | AJ582417 | AJ582474 | AY015773, AY015774 | _ |
| *L. oxytrichum* Sprague^1^ | Australia, Northern Territorium / National BG Canberra, Australia / s.n. | AJ582424 | AJ582481 | AY015775, AY015776 | _ |
| *L. papillosum* F. Muell.^1^ | Australia, Victoria, Red Cliffs, Bottle Bend River / T.H. Browne, Australia / s.n. | AJ582425 | AJ582482 | AY015777, AY015778 | _ |
| *L. pedicellosum* F. Muell.^2^ | Australia / PERTH, Australia, PRP 641 / s.n. | AM991745 | AM991746 | AY015779, AY015780 | _ |
| *L. perfoliatum* L.^2^ | BG Vácrátót, Hungary / BG Vácrátót, Hungary / s.n. | AM991747 | AM991748 | AY015781, AY015782 | _ |
| *L. phlebopetalum* (F. Muell.) F. Muell.^2^ | Australia / Kings Park & BG, Perth, Australia / S 446-93 | FM178556 | FM178556 | AY015783, AY015784 | _ |
| *L. pholidogynum* F. Muell.^2^ | Australia / PERTH, Australia, PRP 640 / s.n. | AM991749 | AM991750 | AY015785, AY015786 | _ |
| *L. pinnatifidum* Ledeb.^1^ | USA, California, Yolo Co. / Tucker Herbarium, Univ. of California, Davis, 96441 / A. M. Shapiro | AJ582464 | AJ582512 | AY015787, AY015788 | _ |
| *L. pinnatum* Thunb.^1^ | South Africa, Cape Province, Drinkriver farm / PRE, 95056/45 / K. A. Dahlstrand | AJ582439 | AJ582496 | AY015827 | _ |
| *L. pseudohyssopifolium* Hewson^1^ | Australia, Victoria, Mitre Rock, near Mt. Arapiles / La Trobe Univ., Australia / N. H. Scarlett s.n. | AJ582431 | AJ582488 | AY015789, AY015790 | _ |
| *L. pseudopapillosum* Thell.^1^ | Australia, Victoria, Kamarooka Forest / La Trobe Univ., Australia / N. H. Scarlett et al. s.n. | AJ582423 | AJ582480 | AY015791, AY015792 | _ |
| *L. pseudotasmanicum* Thell.^1^ | Australia, Victoria, George National Park / La Trobe Univ., Australia s.n. / N. H. Scarlett et al. | AJ582432 | AJ582489 | AY015826 | _ |
| *L. quitense* Turcz.^1^ | Ecuador, Prov. Tungurakua, road from Pillaro to Ambato / MO, 3792042 / C. E. & M. Ceron | AJ582463 | AJ582511 | AY015793, AY015794 | _ |
| *L. rigidum* Pomel^2^ | Algerien, Teniet-el-Haad / F. Hellwig, Göttingen, Germany / s.n. | AM991755 | AM991756 | AY015828 | _ |
| *L. ruderale* L.^1^ | Germany, Borgholzberg near Oldenburg / BG Oldenburg, Germany, s.n. / s.n. | AJ582465 | AJ582513 | AY015795, AY015796 | _ |
| *L. sativum* L.^1^ | Denmark, Jersie / BG Copenhagen, Denmark, s.n. / s.n. | AJ582459 | AJ582507 | AY015828 | _ |
| *L. schinzii* Thell.^1^ | South Africa, Orange Free State Excelsior, Korannaberg / PRE, 95056/6 / J. du Perez | AJ582440 | AJ582497 | AY015797, AY015798 | _ |
| *L. serra* H. Mann^1^ | Hawaii, Kauai, Kalalau valley near Puu O Kila / National Tropical B.G. Lawai, Hawaii, Kauaii, 915398 / S.P. Pearlman | AJ582450 | AJ582518 | AY015799, AY015800 | _ |
| *L. sisymbrioides* ssp. *kawarau* (Petrie) Thell.^1^ | New Zealand, Central Otago, Slapjack Creek / P. de Lange, Dept. Conserv., Auckland Conservancy, New Zealand, 950766 / R. B. Allen | AJ582419 | AJ582476 | AY015801, AY015802 | _ |
| *L. sisymbrioides* ssp.*matau* (Petrie) Thell.^1^ | New Zealand, Central Otago, Galloway / P. de Lange, Dept. Conserv., Auckland Conservancy, New Zealand , 950767 / R. B. Allen | AJ582418 | AJ582475 | AY015803, AY015804 | _ |
| *L. sisymbrioides* Hook.f. ssp. *sisymbrioides*^1^ | New Zealand, Central Otago, Pisa Flats / P. de Lange, Dept. Conserv., Auckland Conservancy, New Zealand , 950768 / R. B. Allen | AJ582420 | AJ582477 | AY015805, AY015806 | _ |
| *L. spinescens* DC.^1^ | Israel, Upper Galilee / BG Univ. Tel Aviv, Israel, s.n / s.n. | AJ582461 | AJ582509 | AY015807, AY015808 | _ |
| *L. spinosum* Ard.^1^ | Turkey, Central Anatolia, near Gaziantep / ETSIA, 436-6229-83 / s.n. | AJ582460 | AJ582508 | AY015824 | _ |
| *L. tayloriae* Al-Shezbaz^2^ | Chile, Atacama Region, Copiapo Prov. / MO, 4355023 / C.M. Taylor et al. 10804 | AM991765 | AM991766 | AM991832 | _ |
| *L. tenuicaule* Kirk^1^ | New Zealand, Kakanui, Shag Point / P. de Lange, Dept. Conserv., Auckland Conservancy, New Zealand, 950191 / P. de Lange | AJ582421 | AJ582478 | AY015809, AY015810 | _ |
| *L. thurberi* Wooton^2^ | USA / Univ. of California, Davis 40791 / D. Miller | AM991767 | AM991768 | AM991833 | _ |
| *L. trifurcum* (Sond.) Marais^1^ | South Africa, Cape Province, SW slopes of Pakhuispiek, cedarberg / PRE, 95056-111 / H. C. Taylor / s.n. | AJ582438 | AJ582495 | AY015811, AY015812 | _ |
| *L. villarsii* Gren. & Godron ssp. *reverchonii* (Deb.) Breist.^2^ | Spain, Guadalajara / Ministerio de Agricultura Pesca y Alimentacion, Spain, 7015-86 / s.n. | AM991771 | AM991772 | AY015825 | _ |
| *L. virginicum* L.^3^ | Mexico, Carrizal Chico, riverbed / Univ. of Osnabrück, Germany / R.Stöckmann & K. Bosbach, s.n. | AF283496 | AF283497 | AY015813, AY015814 | _ |
| *Cardaria draba* (L.) Desv. ssp. *chalepensis* (L.) O.E. Schulz^2^ | Iran, Karadj / ETSIA, 168-3706-75 / s.n. | AM991773 | AM991774 | AY015695, AY015696 | _ |
| *Cardaria pubescens* (C.A. Mey.)^4^ | USA, Wyoming / MO, 4005756 / s.n. | AJ628279 | AJ628280 | AY015697, AY015698 | _ |
| *Coronopus didymus* (L.) Sm.^4^ | France, Brittany, Pabu / BG of Potsdam, Index 1995/96, 1745 / s.n. | AM991775 | AM991776 | AM991834, AM991835 | _ |
| *Coronopus integrifolius* (DC.) Spreng.^2^ | South Africa, Cape Prov. / PRE, 95056/231 / N. Jürgens 28005 | AM991777 | AM991778 | AM991836, AM991837 | _ |
| *Coronopus niloticus* (Del.) Spreng.^2^ | Sudan, Jebel Aulia / WAG, 0143228 / G.J.H. Amshoff s.n. | AM991781 | AM991782 | AM991840 | _ |
| *Coronopus rhytidocarpus* Makl^2^ | Argentina, Tierra del Fuego / Brassicaceae collection, Osnabrück Univ / B. Neuffer s.n. | AM991783 | AM991784 | AM991841, AM991842 | _ |
| *Coronopus serratus* Desv.^2^ | Argentina, Prov. Corrientes / MWC 2320 / Troels Myndel Pedersen 1766 | AM991785 | AM991786 | AM991843, AM991844 | _ |
| *Coronopus squamatus* (Forssk.) Asch^4^ | Portugal, Aljezur (Odeceixe) / BG of Lisbon, Index 1994, No. 239 / s.n. | AJ628287 | AJ628288 | AM991845, AM991846 | AM991938 |
| *Coronopus violaceus* (Munby) Kuntze^4^ | Morocco, N. Azrou / INIA Madrid, Index 1990, No. 206 / s.n. | AJ628289 | AJ628290 | AM991847, AM991848 | AM991939 |
| *Stroganowia affghana* (Boiss.) Pavlov^2^ | Iran / MO / K.H. Rechinger 53578 | AM991787 | AM991788 | AM991849, AM991850 | _ |
| *Stroganowia brachyota* Kar. & Kir.^2^ | Kazakhstan Tian-Shan / MW / s.n. | AM991791 | AM991792 | AM991851 | _ |
| *Stroganowia litwinowii* Lipskyi^2^ | Turkmenistan / MO / V.V. Nykitin s.n. | AM991795 | AM991796 | AM991853, AM991854 | _ |
| *Stroganowia sagittata* Kar. & Kir.^2^ | Kazakhstan / OSBU 04-0307-50-00 / s.n. | AM991799 | AM991800 | AM991856 | _ |
| *Winklera patrinoides* Regel^2^ | Turkmenistan, Terra Kulab / UPS / s.n. | AM991811 | AM991812 | AM991860 | _ |
| *Winklera silaifolia* Korsh.^2^ | Tajikistan, Lake Kizilkul / MW / s.n. | AM991813 | AM991814 | AM991861 | _ |
| *Hornungia petraea* (L.) Reichenbach^4^ | Italy, Alto Adige / Univ. Osnabrück, Germany / s.n. | AJ628293 | AJ628294 | AY015699, AY015700 | _ |

^1^ Sequences taken of taxa from Mummenhoff et al. 2003

^2^ Sequences taken of taxa from Mummenhoff et al. 2009

^3^ Sequences taken of taxa from Mummenhoff et al. 2001

^4^ Sequences taken of taxa from Mummenhoff et al. 2004

**References**

1. Mummenhoff K, Linder P, Friesen N, Bowman JL, Lee JY and Franzke A. Molecular evidence for bicontinental hybridogenous genomic constitution in *Lepidium* sensu stricto (Brassicaceae) species from Australia and New Zealand. Am J Bot. 2003;91: 254-61.
2. Mummenhoff K, Polster A, Muhlhausen A and Theissen G. *Lepidium* as a model system for studying the evolution of fruit development in Brassicaceae. J Exp Bot. 2009;60: 1503-1513.
3. Mummenhoff K, Coja U and Brueggemann H. *Pachyphragma* and *Gagria* (Brassicaceae) revisited: Molecular data close relationship to *Thlaspi* s.str. Folia Geobot. 2001;36: 293-302.
4. Mummenhoff K and Muehlhausen A. Molekulare Analysen zur Systematik der Tribus Heliophileae (Brassicaceae) in Sued-Afrika. Unpublished; 2004.
