# Supplementary material for: Pre-Holocene Origin for the Coronopus navasii Disjunction: Conservation Implications from Its Long Isolation
Source: PLoS One. 2016 Jul 27;11(7):e0159484. doi: 10.1371/journal.pone.0159484 (PMC4963129; doi:10.1371/journal.pone.0159484)
Supplement: S2 Table — (DOCX) [file pone.0159484.s007.docx]

**S2 Table. List of the studied material of Malvidae used for the divergence age estimate.**

| **Taxón** | **Origin / Source / Collector** | **GenBank accession number (matK)** | **Cite** |
| --- | --- | --- | --- |
| *Aethionema grandiflora* L. | Germany / Botanic Garden Jena / s.n. | AF144354 | Koch et al (2001) |
| *Alliaria petiolata*L. | Thüringen, Jena, Germany / _ / leg. KOCH | AF144363 | Koch et al (2001) |
| *Anisoptera marginata* Korth. | _ / K / Chase 2486 | AJ581409 | Hilu et al (2003) |
| *Apophyllum anomalum*F. Muell. | _ / MO / Covery 12044 | AY483227 | Hall et al (2004) |
| *Arabidopsis lyrata* (L.) O’Kane and Al-Shehbaz ssp. *lyrata* | Bash-Bish, Birkshire Cty, MA, USA / _ / leg.MITCHELL-OLDS | AF144342 | Koch et al (2001) |
| *Arabis glabra* (L.) Bernh | Niedersteinbeck, NRW, Germany / _ / leg. KOCH | AF144333 | Koch et al (2001) |
| *Armoracia rusticana* G.Gaertn., B.Mey. & Scherb. | _ / _ / _ | FN597648 | Bakker et al (2009) |
| *Batis maritima* L. | _ / WIS / Iltis 30500 | AY483219 | Hall et al (2004) |
| *Bixa orellana* L. | _ / BONN / A. Worberg 040 | FM179929 | Worberg et al (2009) |
| *Bombax buonopozense* P.  Beauv | _ / WIS / Alverson s.n. | AY321171 | Nyffeler et al (2005) |
| *Brassica napus* L. | _ / IPK (Institute of Plant Genetics and Crop Plant Research, Germany), CR162-95 / _ | AB354273 | Lu et al (2008) |
| *Brestchneidera sinensis* Hemsl. | _ / WIS / Leu & Lin 726 | AY483220 | Hall et al (2004) |
| *Cadaba virgata* Boj. | _ / MO / Lewis et al. 534 | EU371753 | Hall (2008) |
| *Cakile maritima* Scop. | _ / MO / Beilstein 01-76 | GQ424577 | Couvreur et al (2010) |
| *Capparis spinosa* L. | _ / K / Chase 2751 | AY491650 | Hilu et al (2003) |
| *Capsella bursa-pastoris* L. | _ / _ / _ | HQ619802 | Ferri et al (2008) |
| *Cardamine hirsuta* L. | _ / _ / _ | HQ619803 | Ferri et al (2008) |
| *Cardaria draba* (L.) Desv. | Azores / BM / H. Schaefer 2008-295 | HM850735 | Schaefer et al (2011) |
| *Carica papaya* L. | _ / _ / Chase 2508 | AY042564 | Cuenoud et al (2002) |
| *Caylusea latifolia* P. Taylor. | Eritrea, Asmera town / UPS / O. Ryding 1486 | GQ891209 | Martín-Bravo et al (2010) |
| *Cleome hassleriana* Chodat | _ / K / Chase 3015 | AY491649 | Hilu et al (2003) |
| *Cleome pilosa* Benth. | _ / WIS / Iltis 30585 | AY483231 | Hall et al (2004) |
| *Cleome viridiflora* Schreb. | _ / MO / Solomon s.n. | AY483232 | Hall et al (2004) |
| *Cochlearia danica* L. | Borkum, Lower Saxony, Germany / _ / Leg. KOCH | AF174531 | Koch et al (2001) |
| *Coronopus squamatus*(Forssk.) Asch. | Azores / BM / H. Schaefer 2008-526 | HM850736 | Schaefer et al (2011) |
| *Crateva palmeri* Rose | _ / WIS / Hall 105 | AY483229 | Hall et al (2004) |
| *Cylicomorpha parviflora* Urban | Nairobi, Kenya / _ / Muasy A.M. | AY461575 | Kyndt et al (2005) |
| *Daphne bholua* Buch. | _ / BONN / A. Worberg 026 | FM179927 | Worberg et al (2009) |
| *Descurainia sophia* (L.) Webb ex Prantl | _ / MO / Beilstein 01-19 | GQ424581 | Couvreur et al (2010) |
| *Dombeya spectabilis* Boj. | _ / WIS / Alverson 4008 | AY321173 | Nyffeler et al (2005) |
| *Erophila verna* (L.) DC. | _ / _ / _ | HQ619804 | Ferri et al (2008) |
| *Erysimum handel-mazzettii* Polatschek | China, Yunnan, Zhongdian / KUN / Yue0 369 | DQ409262 | Yue et al (2006) |
| *Floerkea proserpinacoides* Willd. | _ / MICH / Reznicek 8609 | EU002178 | Wang et al (2009) |
| *Forchhammeria trifoliata* Radlkofer | _ / WIS / Hansen 3002 | AY483245 | Hall et al (2004) |
| *Gerrardina foliosa* Oliv. | _ / KEW / Balkwill et al. 11983 | FM179924 | Worberg et al (2009) |
| *Gyrostemon thesioides* (J.D. Hook) A.S. George. | Australia, Naraacorte / MA / C.R. Alcock 3115 | FJ212199 | Martín-Bravo et al (2009) |
| *Halimium lasianthum* (Lam.) Spach lasianthum | Málaga, Spain / _ / P. Vargas 3PV06 | GQ281698 | Guzman et al (2009) |
| *Halimolobos jaegeri* (Munz) Rollins | _ / _ / _ | DQ406763 | Oyama et al (2008) |
| *Helianthemum scopulicola* L. | cultivated / MA / B. Guzmán 67BGA04 | DQ092970 | Guzman & Vargas (2005) |
| *Heliophila variabilis* Burch. ex DC. | _ / _ / _ | GQ424588 | Couvreur et al (2010) |
| *Hibiscus syriacus* L. | USA / cultivated (TENN) / R. Small s.n. | EF207270 | Koopman & Baum (2008) |
| *Hornungia petraea* Rchb. | _ / NMW, NMW3528 / _ | JN893991 | de Vere et al (2012) |
| *Iberis amara* L. | _ / _ / Mummenhoff 1695 | GQ424589 | Couvreur et al (2010) |
| *Isatis tinctoria*L. | Tajikistan / IPK (Institute of Plant Genetics and Crop Plant Research, Germany), (ISA 23/03) / _ | AB354278 | Lu et al (2008) |
| *Jacaratia digitata* (Poeppig et Endl.) Solms.-Laubach | Zamora, Ecuador / RUG36, GENT / Romeijin-Peeters E.H. et Romero J.P. | AY461574 | Kyndt et al (2005) |
| *Koeberlinia spinosa* Zucc. | _ / MO / Al Shehbaz s.n. | AY483222 | Hall et al (2004) |
| *Lepidium apetalum* Willd. | _ / KUN / SCSB-HNJ-0228 | JF954301 | Li et al (2011) |
| *Lepidium campestre* (L.) W.T.Aiton | Ontario, Canadá / "University / of Guelph Herbarium, OAC 89379" / AP464 | HQ593342 | Burgess et al (2011) |
| *Lepidium heterophyllum* (DC.) Bentham | _ / NMW, NMW3532 / _ | JN894631 | de Vere et al (2012) |
| *Lepidium latifolium* L. | _ / NMW, NMW3534 / _ | JN894790 | de Vere et al (2012) |
| *Lepidium perfoliatum* L. | _ / _ / _ | DQ406766 | Oyama et al (2008) |
| *Lepidium ruderale* L. | _ / KUN / LiuJQ0149 | JF954308 | Li et al (2011) |
| *Luehea seemannii* Triana & Planch. | _ / BCI 735189 / _ | GQ982036 | Kress et al (2009) |
| *Maerua kirkii (*Oliv.) F. White | _ / WIS / Hall 261 | AY483230 | Hall et al (2004) |
| *Moringa oleifera* Lam. | _ / WIS / Iltis 30500 | AY483223 | Hall et al (2004) |
| *Nasturtium officinale* R.Br. | _ / WIS / Stahmann 233 | AY483225 | Hall et al (2004) |
| *Neslia paniculata* (L.) Desv. | _ / _ / _ | DQ406767 | Oyama et al (2008) |
| *Noccaea cochleariformis* (DC.) A.Love & D.Love | _ / MO / Beilstein 01-21 | GQ424598 | Couvreur et al (2010) |
| *Ochradenus baccatus* Delile. | Somalia, Mait/ UPS / M. Thulin 4351 | GQ891194 | Martín-Bravo et al (2010) |
| *Oligomeris linifolia* MacBride | _ / WIS / Ertter 5613 | AY483240 | Hall et al (2004) |
| *Peddiea africana* Plant ex Meisn | _ / _ / _ | FJ572800 | Motsi et al (2010) |
| *Pentadiplandra brazzeana* Baill. | _ / WIS / Hall 263 | AY483239 | Hall et al (2004) |
| *Podandrogyne decipiens* (Triana & Planch.) Woodson | _ / WIS / G. Mora 380 | EU371815 | Hall (2008) |
| *Raphanus landra* Moretti ex DC. | Russia / IPK (Institute of Plant Genetics and Crop Plant Research, Germany), RA230-80 / _ | AB354265 | Lu et al (2008) |
| *Reseda crystallina* Webb & Berthel. | Spain, Canary Islands, Fuerteventura / LD / S. Snogerup 16461 | FJ212200 | Martín-Bravo et al (2009) |
| *Reseda lutea* L. | _ / WIS / Rodman 535 | AY483241 | Hall et al (2004) |
| *Reseda luteola* L. | Greece, Crete / UPOS / S. Martín-Bravo 391SMB05 | FJ212206 | Martín-Bravo et al (2009) |
| *Rorippa islandica* (Oeder) Borbás | _ / _ / _ | DQ406770 | Oyama et al (2008) |
| *Sesamoides purpurascens* (L.) G. López. | Spain, Seville, El Castillo de las Guardas / UPOS / S. Martín-Bravo 36SMB04 | FJ212208 | Martín-Bravo et al (2009) |
| *Sisymbrium irio* L. | _ / SENDAI Arabidopsis Seed Center / JOS18 | AF144366 | Koch et al (2001) |
| *Stanleya pinnata* (Pursh) Britton | California, USA / HUH (Harvard University Herbaria) / 1735-69 | JN585005 | Hall et al (2011) |
| *Sterculia tragacantha* Lindl. | _ / WIS / Alverson 4011 | AY321178 | Nyffeler et al (2005) |
| *Tapiscia sinensis* Oliver | _ / K / M. W. Chase 1201 | EU002190 | Wang et al (2009) |
| *Tersonia cyathiflora* (Fenzl) A.S.George | _ / Cranfield PERTH no. 02068682 / _ | AY483238 | Hall et al (2004) |
| *Thlaspi arvense* L. | Hagen a.T.W., Lower Saxony, Germany / _ / leg. KOCH | AF144360 | Koch et al (2001) |
| *Thymelaea hirsuta* Endl. | _ / K / M. W. Chase 1882 | EU002191 | Wang et al (2009) |
| *Tovaria pendula* Ruiz. & Pav. | _ / WIS / Smith and Smith 1834 | AY483242 | Hall et al (2004) |
| *Tropaeolum majus* L. | _ / WIS/ Rodman 529 | AY483224 | Hall et al (2004) |
| *Vella pseudocytisus* L. subsp. *paui* Gómez-Campo | _ / K, ABH / Mateo 5-V-9 | GQ248209 | Hollingsworth et al (2009) |

**References**

Bakker FT, Abdel-Khalik K, Copini P, Wieringa JJ, Mummenhoff K. A *mat*K-based phylogeny of the Brassicaceae. Unpublished. 2009.

Burgess KS, Fazekas AL, Kesanakurti PR, Graham SW, Husband BC, Newmaster SG et al. Discriminating plant species in a local temperate flora using the rbcL + matK DNA barcode. Methods Ecol Evol. 2011;2: 333-340.

Couvreur TL, Franzke A, Al-Shehbaz IA, Bakker FT, Koch MA, Mummenhoff K. Molecular phylogenetics, temporal diversification, and principles of evolution in the mustard family (Brassicaceae). Mol Biol Evol. 2010;27: 55-71.

Cuenoud P, Savolainen V, Chatrou LW, Powell M, Grayer RJ, Chase MW. Molecular phylogenetics of Caryophyllales based on nuclear 18S rDNA and plastid *rbc*L, *atp*B, and *mat*K DNA sequences. Am J Bot. 2002;89: 132-144.

de Vere N, Rich TCG, Ford CR, Trinder SA, Long C, Moore CM et al. DNA Barcoding the Native Flowering Plants and Conifers of Wales. PLoS ONE 7: e37945; 2012. doi:10.1371/journal.pone.0037945.

Ferri G, Alu M, Corradini B, Angot A, Beduschi G. Land plants identification in forensic botany: Multigene barcoding approach. Forensic Sci Int Genet. 2008;593-595.

Guzman B, Lledo MD, Vargas P. Adaptive radiation in mediterranean *Cistus* (cistaceae). PLoS ONE 4: e6362; 2009.

Guzman B, Vargas P. Systematics, character evolution, and biogeography of *Cistus* L. (Cistaceae) based on ITS, trnL-trnF, and matK sequences. Mol Phylogenet Evol. 2005;37: 644-660.

Hall JC. Systematics of Capparaceae and Cleomaceae: an evaluation of the generic delimitations of *Capparis* and *Cleome* using plastid DNA sequence data. Botany.  2008;86: 682-696.

Hall JC, Iltis HH, Sytsma KJ. Molecular phylogenetics of core Brassicales, placement of orphan genera *Emblingia*, *Forchhammeria*, and *Tirania*, and character evolution. Systematic Botany. 2004;29: 654-669.

Hall JC, Tisdale TE, Donohue K, Wheeler A, Al-Yahya MA, Kramer EM. Convergent evolution of a complex fruit structure in the tribe Brassiceae (Brassicaceae). Am J Bot. 2011;98: 1989-2003.

Hilu KW, Borsch T, Muller K, Soltis DE, Soltis PS, Savolainen V et al. Angiosperm phylogeny based on matK sequence information. Am J Bot. 2003;90: 1758-1776.

[Hollingsworth](http://europepmc.org/search/?scope=fulltext&page=1&query=AUTH:%22Hollingsworth%20PM%22) PM, Forrest LL, Spouge JL, Hajibabaei M, Ratnasingham S, et al. A DNA barcode for land plants. Proc Natl Acad Sci U S A. 2009;106: 12794–12797.

Koch M, Haubold B, Mitchell-Olds T. Molecular systematics of the Brassicaceae: evidence from coding plastidic matK and nuclear Chs sequences. Am J Bot. 2001;88: 534-544.

Koopman MM, Baum DA. Phylogeny and Biogeography of Tribe Hibisceae (Malvaceae) on Madagascar. Systematic Botany. 2008;33: 364-374.

Kress WJ, Erickson DL, Jones FA, Swenson NG, Perez R, Sanjur O et al. Plant DNA barcodes and a community phylogeny of a tropical forest dynamics plot in Panama. Proc Natl Acad Sci U S A. 2009;106: 18621-18626.

Kyndt T, Van Droogenbroeck B, Romeijn-Peeters E, Romer -Motochi JP, Scheldeman X, Goetghebeur P et al. Molecular phylogeny and evolution of Caricaceae based on rDNA internal transcribed spacers and chloroplast sequence data. Mol Phylogenet Evol. 2005;37: 442-459.

Li DZ, Gao LM, Li HT, Wang H, Ge XJ, Liu JQ et al. Comparative analysis of a large dataset indicates that internal transcribed spacer (ITS) should be incorporated into the core barcode for seed plants. Proc Natl Acad Sci U S A. 2011;108: 19641-19646.

Lu N, Yamane K, Ohnishi O. Genetic diversity of cultivated and wild radish and phylogenetic relationships among *Raphanus* and *Brassica* species revealed by the analysis of trnK/matK sequence. Breed Sci. 2008;58: 15-22.

Martin-Bravo S, Valcarcel V, Vargas P, Luceño M. Geographical speciation related to Pleistocene range shifts in the western Mediterranean mountains (*Reseda* sect. Glaucoreseda, Resedaceae). Taxon. 2010;59: 466-482.

Martin-Bravo S, Vargas P, Luceño M. Is *Oligomeris* (Resedaceae) indigenous to North America? Molecular evidence for a natural colonization from the Old World. Am J Bot. 2009;96: 507-518.

Motsi MC, Moteetee AN, Beaumont AJ, Rye BL, Powell MP, Savolainen V et al. A phylogenetic study of *Pimelea* and *Thecanthes* (Thymelaeaceae): evidence from plastid and nuclear ribosomal DNA sequence data. Aust Syst Bot. 2010;23: 270-284.

Nyffeler R, Bayer C, Alverson WS, Yen A, Whitlock BA, Chase MW, Baum DA. Phylogenetic analysis of the Malvadendrina clade (Malvaceae s.l.) based on plastid DNA sequences. Org Divers Evol. 2005;5: 109-123.

Oyama RK, Clauss MJ, Formanova N, Kroymann J, Schmid KJ, Vogel H et al. The shrunken genome of *Arabidopsis thaliana*. Plant Syst Evol. 2008;273: 257-271.

Schaefer H, Hardy OJ, Silva L, Barraclough TG, Savolainen V. Testing Darwin's naturalization hypothesis in the Azores. Ecol Lett. 2011;14: 389-396.

Wang H, Moore MM, Soltis PS, Bell CD, Brockington SF, Alexandre R et al. Rosid radiation and the rapid rise of angiosperm-dominated forests. Proc Natl Acad Sci U S A. 2009;106: 3853-3858.

Worberg A, Alford MH, Quandt, Borsch T. Huerteales sister to Brassicales plus Malvales, and newly circumscribed to include *Dipentodon*, *Gerrardina*, *Huertea*, and *Tapiscia*. Taxon. 2009;58: 468-478.

Yue JP, Sun H, Al-Shehbaz IA, Li JH. Support for an expanded solms-laubachia (Brassicaceae): evidence from sequences of chloroplast and nuclear genes. Ann Mo Bot Gard. 2006;93: 402-411.
