# Supplementary material for: Pre-Holocene Origin for the Coronopus navasii Disjunction: Conservation Implications from Its Long Isolation
Source: PLoS One. 2016 Jul 27;11(7):e0159484. doi: 10.1371/journal.pone.0159484 (PMC4963129; doi:10.1371/journal.pone.0159484)
Supplement: S3 Table — Bayes Factors for the competing hypotheses are provided. Difference in the Likelihood between the competing hypotheses and the best topology as well as the p-values obtained from the AU test are given. Asterisks indicate evidence in favor to that hypothesis for the BFs and not rejected hypotheses in the AU tests. (DOCX) [file pone.0159484.s008.docx]

**S3 Table. Results from the** Bayes Factor (BF) and Approximate Unbiased (AU) **hypothesis test analyses.** Bayes Factors for the competing hypotheses are provided. Difference in the Likelihood between the competing hypotheses and the best topology as well as the p-values obtained from the AU test are given. Asterisks indicate evidence in favor to that hypothesis for the BFs and not rejected hypotheses in the AU tests.

| **BAYES FACTOR ANALYSES** | | |
| --- | --- | --- |
|  | ITS | *trn*T-*trn*L |
| ITS | _ | 4670,13 |
| *trn*T-*trn*L | -4679,13* | _ |
| **APPROXIMATE UNBIASED TEST** | | |
|  | ITS | trnT-trnL |
| ITS | -5207* (p=0,24) | -7692,92 (p=0) |
| *trn*T-*trn*L | -48161,1 (p=0) | -4134,57* (p=0,24) |
