# Supplementary material for: Pre-Holocene Origin for the Coronopus navasii Disjunction: Conservation Implications from Its Long Isolation
Source: PLoS One. 2016 Jul 27;11(7):e0159484. doi: 10.1371/journal.pone.0159484 (PMC4963129; doi:10.1371/journal.pone.0159484)
Supplement: S4 Table — (DOCX) [file pone.0159484.s009.docx]

**S4 Table. Mean + SD of AUC scores for the six algorithms used in distribution models.**

| **Algorithm** | **Mean ± SD AUC** |
| --- | --- |
| **ANN** | **0.8155 ± 0.172** |
| **CTA** | **0.845 ± 0.125** |
| **GBM** | **0.850 ± 0.171** |
| **GLM** | **0.845 ± 0.127** |
| **MARS** | **0.830 ± 0.172** |
| **RF** | **0.870 ± 0.081** |
